# Effectiveness of Resource Groups for Improving Empowerment, Quality of Life, and Functioning of People With Severe Mental Illness: A Randomized Clinical Trial

**DOI:** 10.1001/jamapsychiatry.2021.2880

**Published:** 2021-10-13

**Authors:** Cathelijn Tjaden, Cornelis L. Mulder, Wouter den Hollander, Stynke Castelein, Philippe Delespaul, Rene Keet, Jaap van Weeghel, Hans Kroon

**Affiliations:** 1 Department of Reintegration and Community Care, Trimbos Institute, Utrecht, the Netherlands; 2 Tranzo Scientific Center for Care and Welfare, Department of Social and Behavioral Sciences, Tilburg University, Tilburg, the Netherlands; 3 Department of Psychiatry, Erasmus University Medical Center, Rotterdam, the Netherlands; 4 Parnassia Psychiatric Institute, Rotterdam, the Netherlands; 5 Lentis Research, Lentis Psychiatric Institute, Groningen, the Netherlands; 6 Faculty of Behavioural and Social Sciences, Department of Clinical Psychology and Experimental Psychopathology, University of Groningen, Groningen, the Netherlands; 7 Rob Giel Research Center, University of Groningen, University Medical Center Groningen, Groningen, the Netherlands; 8 School of Mental Health and NeuroSciences, Maastricht University, Maastricht, the Netherlands; 9 Mondriaan Mental Health Trust, Maastricht/Heerlen, the Netherlands; 10 Department of Community Mental Health, GGZ Noord-Holland-Noord, Heiloo, the Netherlands; 11 Phrenos Centre of Expertise, Utrecht, the Netherlands

## Abstract

**Importance:**

Although the importance of recovery-oriented care for people with severe mental illness (SMI) is widely acknowledged, essential elements such as personalization and involvement of significant others are not adequately implemented in practice.

**Objective:**

To determine whether using resource groups (RGs) within flexible assertive community treatment (FACT) has favorable effects on empowerment and recovery-related outcomes in people with SMI.

**Design, Setting, and Participants:**

This assessor-blind, multisite randomized clinical trial was conducted from September 1, 2017, to September 30, 2020, with follow-up at 9 and 18 months. A total of 158 participants aged 18 to 65 years meeting the criteria for SMI were randomly allocated to FACT plus RG vs FACT as usual (1:1) in 20 FACT teams throughout the Netherlands. Data were analyzed from September 1, 2020, to January 31, 2021. The study was prespecified in the trial protocol and data from the intent-to-treat population were analyzed.

**Interventions:**

In the FACT plus RG condition, patients chose members from their informal and formal networks to form an RG that meets quarterly to discuss self-formulated recovery goals. The RG was integrated into the multidisciplinary support provided by the FACT team. In the FACT as-usual condition, empowerment (defined as overcoming powerlessness and gaining control of one’s life) and involvement of significant others was also part of the provided care, but without the structure of the RG.

**Main Outcomes and Measures:**

The primary outcome was self-reported empowerment, measured with the Netherlands Empowerment List.

**Results:**

A total of 158 participants with SMI (median age, 38 [median absolute deviation, 13] years; 93 men [58.9%]) were randomized to FACT plus RG (n = 80) or FACT as usual (n = 78) care. Intention-to-treat analyses showed that randomization to the RG condition was associated with a clinically significant increase in empowerment (Cohen *d*, 0.54; 95% CI, 0.21-0.86) and improved outcomes with small to medium effect sizes in terms of quality of life (Cohen *d*, 0.25; 95% CI, −0.07 to 0.56), personal recovery (Cohen *d*, 0.38; 95% CI, 0.06-0.69), quality of social contact (Cohen *d*, 0.24; 95% CI, −0.07 to 0.56), disability (Cohen *d*, 0.29; 95% CI, −0.03 to 0.60), general functioning (Cohen *d*, 0.30; 95% CI, −0.01 to 0.62), and social functioning (Cohen *d*, 0.28; 95% CI, −0.04 to 0.59). No differences between conditions were found regarding psychopathological symptoms, attachment, frequency of social contact, and employment. Compared with FACT as usual, participants who stayed with the assigned treatment in the RG condition were more satisfied with treatment at 9 (Cohen *d* = 0.45; *t*_135_ = −2.62; *P* = .009) and 18 (Cohen *d* = 0.41; *t*_116_ = −2.22; *P* = .02) months.

**Conclusions and Relevance:**

These findings show that working with RGs improves empowerment and other mental health outcomes in people with SMI who receive community-based mental health services. This method of network-oriented care empowers people with SMI within their own environment.

**Trial Registration:**

Netherlands Trial Register Identifier: NL6548

## Key Points

**Question:**

Does the structure of a resource group have favorable effects on empowerment and recovery-related outcomes of people with severe mental illness?

**Findings:**

In this randomized clinical trial of 158 people with severe mental illness aged 18 to 65 years, self-reported empowerment improved significantly when working within a resource group method compared with well-established community-based care. Exploratory secondary outcomes such as quality of life and social functioning also indicated a broad range of benefits.

**Meaning:**

These findings suggest that resource groups establish widely endorsed principles of empowerment and engagement of significant others as cornerstones of community-based mental health care.

## Teaser

This randomized clinical trial investigates whether integrating a resource group model into community care has favorable effects on empowerment, quality of life, recovery, social functioning, and symptoms compared with community care as usual among people with severe mental illness.

## Introduction

Severe mental illnesses (SMIs) are psychiatric disorders that imperil lives over a long period, challenging and preoccupying mental health professionals.^1^ Although the potential benefits of involving significant others in mental health care are well documented,^2^ poor implementation rates are often reported.^3,4,5,6^ The resource group (RG) method, which builds on traditions of family intervention and integrated care, is a promising initiative to overcome this problem by providing a structure to ensure that family, friends, and caregivers maintain full involvement in routine services, thereby becoming collaborative partners in the recovery process. A patient’s RG comprises individuals from their informal (ie, friends, family) and formal (ie, social worker, psychiatrist, peer worker) networks and meets quarterly to discuss the patient’s recovery goals and jointly develop a plan for achieving them. By being part of an RG, significant others acquire skills to contribute to the goals, and attention is paid to their burden and needs as well as those of the patient.

Because the RG method designates patients as the directors of their RG, they are encouraged to take ownership in shaping the support that meets their needs and aspirations. The primary aim of the method is to facilitate patients’ empowerment (ie, processes in which someone rediscovers their identity and takes their life in their own hands).^7^ Empowerment is identified as a key aspect of recovery-oriented mental health care in itself,^8,9,10^ but it is also recognized as a mediator toward improved long-term health.^11^ That is, empowered patients are thought to improve their health behaviors in terms of their self-esteem, social and community functioning, and abilities to manage their illness.^11,12,13,14,15^ In addition, a lack of empowerment is related to increased depression and hopelessness^16,17,18,19^ and impaired quality of life.^16,20^ The importance of empowerment in disease prevention and health promotion is recognized in various international policy guidelines.^21,22,23,24^

The origins of the RG method lie in the optimal treatment model, which integrates biomedical, psychological, and social strategies in the management of SMI.^25,26^ In Sweden, the optimal treatment model was further developed to RG Assertive Community Treatment (RACT).^27,28^ A meta-analysis showed outcomes in favor of RACT for patients with psychosis in social functioning, well-being, and symptoms.^28^ However, RACT consists of various differences from care as usual, and the use of RGs was only one of these. The present study therefore investigated whether integrating RGs with community care has favorable effects on empowerment as well as quality of life, recovery, social and societal functioning, and symptoms compared with community care as usual.

## Methods

### Participants and Design

This assessor-blinded, 2-arm, pragmatic randomized clinical trial was conducted from September 1, 2017, to September 30, 2020, within the context of flexible assertive community treatment (FACT),^29,30,31^ the community-based care of choice for people with SMI in the Netherlands. The study was prespecified in the trial protocol (Supplement 1),^32^ which was approved by the Medical Ethics Committee at VU University Medical Centre, Amsterdam. No important changes were made after trial approval, and no data were analyzed before study completion and database lock. Participants provided written informed consent after receiving a complete description of the study. The study followed the Consolidated Standards of Reporting Trials (CONSORT) reporting guideline.^33^

Participants were recruited from 20 FACT teams at 9 mental health care centers throughout the Netherlands. Eligibility screening and enrollment were conducted by FACT professionals during the intake phase for new patients. The 6 inclusion criteria consisted of age 18 years or older, an SMI according to the Dutch definition,^34^ estimated FACT team future involvement for at least 12 months of care but not more than 2 years in the past, capacity to provide written informed consent, and sufficient Dutch language skills.

Randomization was performed after baseline assessment. Follow-up assessments were conducted after 9 and 18 months. Assessments took place at a participant’s home or at the treatment site and took 90 to 120 minutes to complete. Owing to the COVID-19 pandemic, 50 of 416 interviews (12.0%) took place by telephone.

### Randomization and Blinding

Patients were randomized to FACT plus RG or FACT as usual (1:1) via an interactive web-response system based on a random allocation sequence generated by a statistician who was not involved in the trial. The system sent an email with the randomization to the involved mental health professionals, who notified the participants. Randomization was stratified by the FACT team. To conceal allocation sequence, random permuted blocks with sizes 2 and 4 were used.

Participants and mental health professionals were not blinded to allocation, whereas the research staff conducting follow-up assessments at 9 and 18 months were blinded. To assess blinding, the research staff filled in control questions about participants’ allocation status after each assessment.

### Interventions

#### Resource Groups

Working with RGs within FACT involved 6 phases (eTable 1 in Supplement 2) to establish an RG that would meet quarterly. The intervention protocol was adapted from the RACT program^27,35^ and is described in detail elsewhere.^32,36^ Briefly, patients drafted their RG plans, which consisted of short- and long-term recovery goals and early warning signs, with the support of a mental health professional who was trained in the RG method. The patients then asked (“nominated”) significant others and/or professionals who could contribute in working toward the goals to join the RG. The composition was flexible and could change according to patients’ goals and phase of recovery.

Together with the mental health professional, each patient prepared for their first RG meeting by setting the agenda and deciding on a location and chairperson. Before the meeting, the professional invited the nominated members of the patient’s RG for an in-depth interview to discuss their involvement and their mutual relationships among RG members. The RG met quarterly to discuss and evaluate the patient’s recovery goals and the plan for achieving them.

Training in the RG method for professionals involved 2 full days and 2 half days and attendance at 6 weekly telephonic supervision meetings in small groups. There were no restrictions regarding educational background.

#### FACT As Usual

FACT as usual consisted of multidisciplinary treatment and care. According to patients’ needs and goals, this included case management, peer support, and psychiatric medication monitoring.^29^ Support and involvement of significant others could be part of treatment but not in the structured approach used in the RG method. Twenty of 22 participating FACT teams (90.9%) were certified to ensure similar and guaranteed quality of care.

#### Model Fidelity

Adherence and model fidelity were assessed with the RG model evaluation tool (eTable 2 in Supplement 2), which was developed in parallel with the study. The RG model evaluation tool consisted of 25 questions that were completed by the professional after each RG meeting from which a model fidelity score per RG was obtained.

### Outcomes

The primary outcome was self-reported empowerment, which was measured using the Netherlands Empowerment List (40 items)^7^ (eTable 6 in Supplement 2). The Netherlands Empowerment List contains 6 subscales: confidence and purpose, self-management, connectedness, social support, professional help, and caring community. Internal consistency (Cronbach α = 0.94), aspects of validity, reproducibility (Cronbach α = 0.79), and responsiveness were good.^7,37^ The questionnaire has been used as an outcome measure in several randomized clinical trials.^38,39,40,41,42^ Secondary self-report outcomes were quality of life (Manchester Short Assessment of Quality of Life^43^), psychopathological symptoms (Brief Symptom Inventory-18^44^), difficulties in adult attachment (Revised Adult Attachment Scale^45^), frequency of social contact (5 Likert scales [range, 1-7]), quality of social contact (5 self-reported Likert scales [range, 1-5]), employment (binary variable: a volunteer or paid job [1] or no job [0]), and satisfaction with treatment (Client Satisfaction Questionnaire^46^) and with involvement of relatives in treatment (subscale of Verona Service Satisfaction Scale–European Version^47^).

Interview outcomes were personal recovery (Individual Recovery Outcomes Counter^48^) and disability (World Health Organization Disability Assessment Schedule-32^49^). Assessor-based outcomes were global functioning (Global Assessment of Functioning^50^) and social functioning (Social and Occupational Functioning Assessment Scale^51^) as scored by blinded investigators. Outcomes were assessed at 0, 9, and 18 months, except for treatment satisfaction measurements (Client Satisfaction Questionnaire and Verona Service Satisfaction Scale–European Version, assessed at 9- and 18-month follow-up).

### Statistical Analyses

Data were analyzed from September 1, 2020, to January 31, 2021, in R, version 3.0+ (R Program for Statistical Computing) and SPSS, version 27.0 (IBM Corporation). Assuming an effect size of Cohen *d* = 0.50,^52^ a 2-sided α = .05, and repeated measurement analysis, a minimum sample size of 133 was required to detect significant differences between groups with a power (β) of 80%.

Outcomes were analyzed according to the intention-to-treat population using repeated-measures linear mixed modeling and the R package lme4 (R Program for Statistical Computing).^53^ Linear mixed modeling includes incomplete cases in the analysis and uses restricted maximum likelihood estimation to calculate parameter estimates. Because linear mixed modeling performs internal imputation, no other procedure of missing data was performed.^54^

Although the data had a 4-level structure (repeated measures, patients, teams, and centers), adding a random intercept for center did not offer a better model fit (χ^2^_8_  = 0.51 [n = 158]; *P* = .91) when compared with the more parsimonious 3-level structure. Hence, intercepts for patients nested in teams were included as random effects. To determine whether outcomes significantly differed between conditions over time, linear mixed models were fitted with the respective outcomes as dependent variables. The independent part consisted of the fixed effect log time and the interaction effect between condition and log time. Treatment effectiveness was determined by comparing the mean slope in the 2 conditions, reflected by and reported as Cohen *d*.^55^ Completers were defined as participants who had attended at least 2 RG meetings, 2 being the median. In addition, we explored whether differences in improvements of secondary outcomes seen between groups are accounted for by an early change in empowerment. To this end, mediation analyses were performed (R package mediation [R Program for Statistical Computing]) with change between times 0 and 1 in empowerment as the mediating variable, treatment as the independent variable, and slope of the respective secondary outcome between times 0 and 2 as the dependent variable.

## Results

A total of 403 people with SMI were screened for eligibility, and 158 participants were randomized to FACT plus RG (n = 80) or FACT as usual (n = 78) (Figure). The baseline group characteristics were similar (Table 1). Participants’ median age was 38 (median absolute deviation [MAD], 13) years; 93 (58.9%) were men and 65 (41.1%) were women. Primary clinical classifications varied, with a similar proportion of comorbidity in the intervention and control conditions (48 of 80 [60.0%] vs 42 of 78 [53.8%], respectively). During 28 of 258 assessments (10.9%), research staff were not blinded to allocation.

**Figure.**
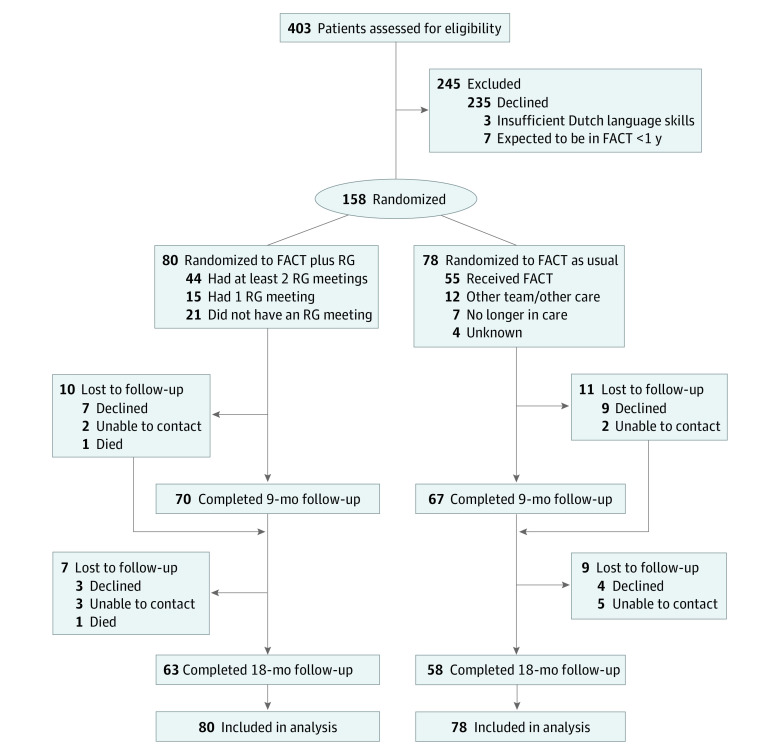
CONSORT Diagram of Participant Flow Through the Trial Patients were recruited from 20 flexible assertive community treatment (FACT) teams (range, 2-18 patients per FACT team) at 9 mental health care centers (range, 1-3 teams per center). RG indicates resource group.

**Table 1.** Baseline Characteristics of the Study Group

Characteristic	Study group^a^		
	All (N = 158)	FACT as usual (n = 78)	FACT plus RG (n = 80)
Sex			
Male	93 (58.9)	46 (59.0)	47 (58.9)
Female	65 (41.1)	32 (41.0)	33 (41.3)
Age, median (MAD), y	38 (13)	41 (12)	37 (14)
Educational attainment			
None or only elementary school/GED	37 (23.4)	20 (25.6)	17 (21.3)
At least secondary education	121 (76.6)	58 (74.4)	63 (78.8)
Partnered			
Yes	53 (33.5)	24 (30.8)	29 (36.3)
No	105 (66.5)	54 (69.2)	51 (63.8)
Employment			
Employed	26 (16.5)	15 (19.2)	11 (13.8)
Volunteer work	24 (15.2)	10 (12.8)	14 (17.5)
Unemployed	79 (50.0)	37 (47.4)	42 (52.5)
Other	29 (18.4)	16 (20.5)	13 (16.3)
Living situation			
Alone	61 (38.6)	36 (46.2)	25 (31.3)
With partner and/or children	46 (29.1)	20 (25.6)	26 (32.5)
With parents	13 (8.2)	9 (11.5)	4 (5.0)
Supported housing	30 (19.0)	11 (14.1)	19 (23.8)
Other	8 (5.1)	2 (2.6)	6 (7.5)
Ethnicity^b^			
Dutch	93 (58.9)	47 (60.3)	46 (57.5)
Western	19 (12.0)	9 (11.5)	10 (12.5)
Nonwestern	45 (28.5)	22 (28.2)	23 (28.8)
Unknown	1 (0.6)	0	1 (1.3)
Lifetime contact with mental health service, median (MAD), y	6 (5.93)	7 (7.41)	5 (5.13)
Lifetime admissions to psychiatric hospital			
Never	41 (25.9)	22 (28.2)	19 (23.8)
1	38 (24.1)	17 (21.8)	21 (26.3)
2-4	73 (46.2)	36 (46.2)	37 (46.3)
>4	6 (3.8)	3 (3.8)	3 (3.8)
Main clinical classification			
Schizophrenia or schizoaffective disorder	36 (22.8)	18 (23.1)	18 (22.5)
Other psychosis	22 (13.9)	15 (19.2)	7 (8.8)
Bipolar affective disorder	12 (7.6)	3 (3.8)	9 (11.3)
Depressive disorder	18 (11.4)	11 (14.1)	7 (8.8)
Anxiety disorder	4 (2.5)	0	4 (5.0)
Posttraumatic stress disorder	5 (3.2)	1 (1.3)	4 (5.0)
Substance-related disorders	23 (14.6)	11 (14.1)	12 (15.0)
Personality disorder	13 (8.2)	7 (9.0)	6 (7.5)
Autism spectrum disorder	8 (5.1)	4 (5.1)	4 (5.0)
Other	6 (3.8)	2 (2.6)	4 (5.0)
Unknown	11 (7.0)	6 (7.7)	5 (6.3)

Abbreviations: FACT, flexible assertive community treatment; GED, General Educational Development; MAD, median absolute deviation; RG, resource group.

^a^ Unless otherwise indicated, data are expressed as number (%) of patients.

^b^ Ethnicity was classified according to the national guidelines of the Central Bureau of Statistics. Nonwestern ethnicity included participants from the former Dutch colony Suriname.

Follow-up data were available by September 30, 2020. Fifty-nine participants (73.8%) allocated to FACT plus RG had at least 1 RG meeting, and 44 (55.0%) had at least 2 RG meetings. Reasons for not starting up an RG were lack of motivation for the patient (n = 6), lack of time or motivation for the mental health professional (n = 3), repeated crisis (n = 8), or referral to other care (n = 4). Model-fidelity scores indicated a sufficient dose of the RG method (mean [SD], 3.99 [0.46]; median, 4.00 [range, 2.71-4.71]) (eTable 3 in Supplement 2). For the 21 participants (26.3%) without an RG meeting, model fidelity was scored as 0.

### Primary and Secondary Outcomes

The primary analysis showed a greater increase in empowerment over time in the FACT plus RG condition than in the FACT as usual condition; this is reflected in a significant treatment-by-time interaction effect (estimate 0.40555 [SD, 0.07543]; *P* < .001) with a moderate effect size (Cohen *d* = 0.54; 95% CI, 0.21-0.86), indicating significantly different slopes between treatment groups (Table 2, Table 3, and eFigures 1 and 2 in Supplement 2).

**Table 2.** Primary and Secondary Outcome Measures During the 3 Time Points by Condition

Outcome	Study group					
	FACT plus RG			FACT as usual		
	Baseline (n = 80)	9-mo Follow-up (n = 70)	18-mo Follow-up (n = 63)	Baseline (n = 78)	9-mo Follow-up (n = 67)	18-mo Follow-up (n = 58)
NEL (primary outcome), mean (SD) score^a^	3.32 (0.51)	3.55 (0.53)	3.77 (0.57)	3.34 (0.52)	3.34 (0.62)	3.38 (0.70)
Secondary outcomes, mean (SD) score						
MANSA^b^	4.12 (0.88)	4.49 (0.74)	4.67 (0.74)	4.26 (0.85)	4.34 (0.90)	4.48 (1.04)
IROC^c^	3.49 (0.77)	4.04 (0.77)	4.21 (0.73)	3.65 (0.73)	3.89 (0.93)	3.98 (1.04)
WHODAS 32^d^	37.73 (18.73)	30.93 (19.31)	29.90 (20.51)	36.62 (19.92)	32.87 (19.99)	32.87 (18.96)
GAF^e^	47.91 (10.22)	53.8 (10.16)	58.33 (11.76)	51.45 (10.58)	54.03 (11.26)	54.84 (13.43)
SOFAS^f^	51.55 (9.49)	55.8 (9.44)	59.13 (10.81)	53.71 (11.46)	54.82 (11.05)	56 (13.96)
BSI^g^	2.11 (0.72)	1.98 (0.86)	1.92 (0.82)	2.15 (0.85)	1.98 (0.81)	1.91 (0.76)
RAAS^h^	2.92 (0.61)	2.84 (0.66)	2.78 (0.68)	2.99 (0.70)	2.85 (0.74)	2.9 (0.74)
Frequent social contact^i^	4.51 (1.15)	4.5 (1.10)	4.5 (0.89)	4.58 (1.11)	4.5 (1.25)	4.24 (1.25)
High-quality social contact^j^	3.76 (0.76)	3.98 (0.58)	3.99 (0.55)	3.76 (0.70)	3.84 (0.68)	3.86 (0.66)
Employment^k^	0.4 (0.49)	0.47 (0.50)	0.52 (0.50)	0.49 (0.50)	0.49 (0.50)	0.52 (0.50)

Abbreviations: BSI, Brief Symptom Inventory; FACT, flexible assertive community treatment; GAF, Global Assessment of Functioning Scale; IROC, Individual Recovery Outcomes Counter; MANSA, Manchester Short Assessment of Quality of Life; NEL, Netherlands Empowerment List; RAAS, Revised Adult Attachment Scale; RG, resource group; SOFAS, Social and Occupational Functioning Scale; WHODAS 32, World Health Organization Disability Assessment Schedule 2.0.

^a^ Scores range from 1 to 5, with higher scores indicating better empowerment.

^b^ Scores range from 1 to 7, with higher scores indicating better quality of life.

^c^ Scores range from 1 to 6, with higher scores indicating better recovery.

^d^ Scores range from 1 to 5, with higher scores indicating more disability.

^e^ Scores range from 0 to 100, with higher scores indicating better functioning.

^f^ Scores range from 0 to 100, with higher scores indicating better social functioning.

^g^ Scores range from 1 to 5, with higher scores indicating more symptoms.

^h^ Scores range from 1 to 5, with higher scores indicating more attachment unsafety.

^i^ Scores range from 1 to 7, with higher scores indicating higher frequency.

^j^ Scores range from 1 to 7, with higher scores indicating better quality of social contact.

^k^ Zero indicates no job; 1, having a volunteer or paid job.

**Table 3.** Outcomes Linear Mixed Model Analyses of Primary and Secondary Outcomes

Outcome and effect	Slope (SD)	Cohen *d* effect size (95% CI)^a^
Primary outcome		
Empowerment (NEL) score^b^		
Control group	0.018 (0.004)	0.54 (0.21 to 0.86)
Treatment group	0.423 (0.003)	
Secondary outcomes		
Quality of life (MANSA) score^c^		
Control group	0.192 (0.007)	0.25 (−0.07 to 0.56)
Treatment group	0.461 (0.007)	
Personal recovery (IROC) score^d^		
Control group	0.276 (0.007)	0.38 (0.06 to 0.69)^e^
Treatment group	0.675 (0.006)	
Disability (WHODAS 32) score^f^		
Control group	−0.079 (0.003)	0.29 (−0.03 to 0.60)
Treatment group	−0.273 (0.003)	
General functioning (GAF) score^g^		
Control group	3.682 (1.540)	0.30 (−0.01 to 0.62)
Treatment group	8.394 (1.465)	
Social and occupational functioning (SOFAS) score^h^		
Control group	2.008 (1.466)	0.28 (−0.04 to 0.59)
Treatment group	6.166 (1.396)	
Symptoms (BSI) score^i^		
Control group	−0.164 (0.005)	0.07 (−0.24 to 0.38)
Treatment group	−0.228 (0.005)	
Attachment (RAAS) score^j^		
Control group	−0.068 (0.004)	0.10 (−0.21 to 0.41)
Treatment group	−0.148 (0.004)	
Frequency of social contact^k^		
Control group	−0.243 (0.014)	0.15 (−0.16 to 0.46)
Treatment group	−0.012 (0.014)	
Quality of social contact^l^		
Control group	0.033 (0.004)	0.24 (−0.07 to 0.56)
Treatment group	0.24 (0.004)	
Employment^m^		
Control group	0.123 (0.132)	0.10 (−0.21 to 0.42)
Treatment group	0.565 (0.128)	

Abbreviations: BSI, Brief Symptom Inventory; GAF, Global Assessment of Functioning Scale; IROC, Individual Recovery Outcomes Counter; MANSA, Manchester Short Assessment of Quality of Life; NEL, Netherlands Empowerment List; RAAS, Revised Adult Attachment Scale; SOFAS, Social and Occupational Functioning Scale; WHODAS 32, World Health Organization Disability Assessment Schedule 2.0.

^a^ Sign and range of effect sizes is adjusted so that positive effect size indicates effects in favor of the flexible assertive community treatment plus resource group condition.

^b^ Scores range from 1 to 5, with higher scores indicating better empowerment.

^c^ Scores range from 1 to 7, with higher scores indicating better quality of life.

^d^ Scores range from 1 to 6, with higher scores indicating better recovery.

^e^ *P* < .001.

^f^ Scores range from 1 to 5, with higher scores indicating more disability.

^g^ Scores range from 0 to 100, with higher scores indicating better functioning.

^h^ Scores range from 0 to 100, with higher scores indicating better social functioning.

^i^ Scores range from 1 to 5, with higher scores indicating more symptoms.

^j^ Scores range from 1 to 5, with higher scores indicating more attachment unsafety.

^k^ Scores range from 1 to 7, with higher scores indicating higher frequency.

^l^ Scores range from 1 to 7, with higher scores indicating better quality of social contact.

^m^ Zero indicates no job; 1, having having a volunteer or paid job.

Exploratory secondary outcomes analyses showed that FACT plus RG was superior to FACT as usual for personal recovery (Individual Recovery Outcomes Counter; Cohen *d*, 0.38 [95% CI, 0.06-0.69]), quality of life (Manchester Short Assessment of Quality of Life; Cohen *d*, 0.25 [95% CI, −0.07 to 0.56]), disability (World Health Organization Disability Assessment Schedule 2.0; Cohen *d*, 0.29 [95% CI, −0.03 to 0.60]), quality of social contact (Cohen *d*, 0.24 [95% CI, −0.07 to 0.56]), general functioning (Global Assessment of Functioning Scale; Cohen *d*, 0.30 [95% CI, −0.01 to 0.62]), and social and occupational functioning (Social and Occupational Functioning Scale; Cohen *d*, 0.28 [95% CI, −0.04 to 0.59]), as reflected by differences in slopes between conditions with a small to moderate Cohen *d* (Tables 2 and 3). With regard to psychopathological symptoms (Brief Symptom Inventory), attachment (Revised Adult Attachment Scale), frequency of social contacts, and employment, there were no differences (Cohen *d*, <0.20) in slopes between the conditions.

### Subgroup Analyses

Exploratory subgroup analyses, in which completers in the FACT plus RG condition (n = 59) were compared with participants in the FACT as usual condition (n = 78), yielded similar or slightly larger effect sizes (eTable 4 in Supplement 2). The effect size for the primary outcome analysis increased from 0.54 to 0.61 (95% CI, 0.28-0.93).

### Mediation Analyses and Treatment Satisfaction

Results from the mediation analyses, as shown in eTable 5 in Supplement 2, revealed that improved empowerment after 9 months was a significant mediator for changes in personal recovery (proportion-mediated standardized mean difference, 0.32; 95% CI, 0.03-0.70; *P* = .04) and general functioning (proportion-mediated standardized mean difference, 0.13; 95% CI, 0.01-0.36; *P* = .04) after 18 months. Compared with the FACT as usual condition, participants in the FACT plus RG condition were more satisfied with their treatment at 9 (Cohen *d*, 0.45; *t*_135_ = −2.62; *P* = .009) and 18 (Cohen *d*, 0.41; *t*_116_ = −2.22; *P* = .02) months as well as the involvement of their relatives at 9 (Cohen *d*, 0.48; *t*_132_ = −3.96; *P* < .001) and 18 (Cohen *d*, 0.59; *t*_115_ = −4.40; *P* < .001) months (Table 4).

**Table 4.** Care Satisfaction

Satisfaction measure	9-mo Follow-up				18-mo Follow-up				
	No. of observations^a^	Mean (SD) score	*t* Test (*df*)	Cohen *d* (95% CI)	No. of observations^a^	Mean (SD) score	*t* Test (*df*)	Cohen *d* (95% CI)
CSQ^b^								
FACT as usual	67	2.89 (0.43)	−2.62 (135)^c^	0.45 (0.11-0.79)	58	2.80 (0.44)	−2.22 (116)^d^	0.41 (0.05-0.77)
FACT plus RG	70	3.09 (0.46)			63	3.01 (0.57)		
VSSS-EU^e^								
FACT as usual	67	3.15 (0.89)	−3.96 (132)^f^	0.48 (0.14-0.82)	58	3.01 (0.74)	−4.40 (115)^f^	0.59 (0.22-0.95)
FACT plus RG	70	3.73 (0.81)			63	3.70 (0.98)		

Abbreviations: CSQ, Client Satisfaction Questionnaire; FACT, flexible assertive community treatment; RG, resource group; VSSS-EU, Verona Service Satisfaction Scale–European Version.

^a^ Uses only available data and without performing imputation.

^b^ Scores range from 1 to 4, with higher scores indicating more satisfaction.

^c^ *P* = .009.

^d^ *P* < .05.

^e^ Indicates relatives’ involvement. Scores range from 1 to 5, with higher scores indicating more satisfaction.

^f^ *P* < .001.

## Discussion

To our knowledge, this randomized clinical trial is the first to examine the effectiveness of RGs for patients with SMI as a way to facilitate empowerment and enhance involvement of significant others. Our results show that empowerment improved significantly when RGs were integrated into FACT compared with FACT as usual. The medium effect size we found is large compared with effect sizes found in other social interventions for people with SMI.^56,57,58^ In addition, FACT plus RG improved quality of life, personal recovery, disability, quality of social contact, and general and social functioning more than FACT as usual. No differences between conditions were found regarding psychopathological symptoms, attachment, frequency of social contact, or employment. At 9 and 18 months, treatment satisfaction was higher in the FACT plus RG group than in the FACT as usual group. Our findings are consistent with those of previous uncontrolled studies of the RG method,^25,28,52^ supporting the use of RGs to improve community-based mental health care for people with SMI.

The strongest effects of the RGs were observed for empowerment, as we expected. A possible explanation for this is offered by the qualitative study on working mechanisms by Tjaden et al,^59^ which showed that when patients were encouraged to be directors of their RG and to think about important aspects of their care—such as whom to involve and which recovery goals to discuss—their ownership concerning illness and recovery was vitalized. This finding reflects the content of the Netherlands Empowerment List and indicates that the RG method made patients feel more confident about their capabilities, such as having meaningful relationships and facing the challenges of their disease.

In addition, within the RG method, significant others were structurally involved, constituting an important difference from the control condition. This may have further strengthened the patients’ belief in their own capabilities. Previous studies^20,60,61^ have shown the importance of family and a social network to the process of empowerment because they facilitate self-esteem and a self-concept of being capable and valuable. The RG method thereby fits within a relational, contextual perspective that underscores the pivotal role of the social context in coping with illness and recovery. Previous studies^62,63^ have recognized that people with SMI often see themselves as inferior and shamed in their relationships with professionals and people from their social networks. Recovering from such imbalance via the openness and joint decision-making processes inherent to the RG meetings may contribute to the empowering effects of the method.^64^

Our findings are in line with those of studies showing that interventions directed toward involving family and social networks are among the most effective for people with SMI.^2,6^ Nevertheless, poor implementation rates for social network involvement are consistently reported.^3,4,5,6^ In this light, most participants with SMI allocated to the intervention condition being able to set up an RG for a longer period of time is noteworthy and suggests that the RG method is a feasible means of providing network-oriented mental health care. However, implementing the method was still challenging, as reflected by the 21 participants who did not have an RG meeting. These implementation difficulties have been described previously^59,64^ and show the importance of investigating barriers and facilitating factors.

The RG method could also be useful for improving collaboration between services. Rather than referring patients to professionals such as employment or housing specialists, continuity in the different phases of illness and recovery can be fostered by inviting these specialists to join the RG meetings.

Overall, our exploratory secondary analyses provide further evidence of the effectiveness of the RG method, although the effect sizes were smaller than those for empowerment. In addition, the mediation analyses showed that a portion of the improvement in several secondary outcomes seen between the 2 groups was accounted for by the change in empowerment. These findings support the notion that empowerment is not a traditional outcome but can be seen as a mediator that functions as an effective strategy for changing an individual’s health behaviors, eventually leading to improved mental health outcomes.^10,11,20^ Empowerment and other clinical outcomes may thus have reciprocally reinforcing influences, and effects might stretch beyond our follow-up times. However, we did not power on the mediation analyses, thus they should be considered exploratory. Future studies with longer follow-up are needed to further investigate how the RG method, empowerment, and other outcomes are related.

We did not detect a significant difference in psychopathological symptoms between conditions. Clinical recovery may be addressed sufficiently within FACT. Medical and psychiatric care are indeed well implemented in FACT, but there are difficulties with services oriented toward recovery and rehabilitation.^65^ Indeed, in FACT as usual, mean empowerment scores did not show change at 18 months. Hence, it seems that the main added value of working with RGs within FACT concerns the functional and personal components of recovery that relate to reengagement in social interactions and a sense of personal agency.

### Strengths and Limitations

A key strength of this study is our comparison of the RG method with an active control intervention with effectiveness that has repeatedly been demonstrated in uncontrolled studies.^66,67,68,69^ Furthermore, the trial was adequately powered and used blinded assessors, and the multisite nature adds to the generalizability of findings.

Our results should also be interpreted in light of some limitations. First, professionals could have been biased during eligibility screening to approach patients who already had a well-functioning social network and/or ability to be an RG director. However, Dutch studies investigating the population with SMI in community-based services report comparable Global Assessment of Functioning Scale scores^70^ and similar demographic and employment characteristics.^71^ Although a comparison with social indicators was not available, this indicates that our study is based on a representative sample. A second limitation is that our model fidelity scale was not validated but designed parallel to the present study. In future research on the validity of the scale, a dose-response association can be deduced to increase our understanding of the effective elements of the method. Third, the experimental condition was performed in the same teams as the control condition. Despite the significant differences between conditions, participants in the control condition may have been exposed to elements of the RG method (ie, spillover). Last, we did not collect any data on potential harms, our power calculation was not based on the secondary outcomes, and 10.9% of the assessors were not blinded during data collection. In addition, participants were not blinded, which may have affected their response on the self-report measurements.

## Conclusions

In this randomized clinical trial, integrating RGs within and into FACT improved empowerment and other mental health outcomes for people with SMI. Replication of the results in various local and international contexts and health economic data are recommended.

## Notes

Supplement 1.
Trial Protocol

Supplement 2. **eTable 1.** The 6 Phases of the Resource Group Method **eTable 2.** Description and Criteria of Scores on the Resource Group Model Evaluation Tool **eTable 3.** Frequency, Mean and SD of Scores on Items of the Resource Group Model Evaluation Tool **eTable 4.** Outcomes Linear Mixed Model Sensitivity Analyses of Primary and Secondary Outcomes of Participants With ≥2 RG Meetings **eTable 5.** Outcomes Mediation Analyses **eTable 6.** The Netherlands Empowerment List (NEL) **eFigure 1.** Trajectory of Mean Empowerment Scores Over Time, Based on the Intention-to-Treat Population **eFigure 2.** Absolute Empowerment Scores Within Participants Across Time Points

Supplement 3.
Data Sharing Statement
