# Supplementary material for: Effectiveness of Resource Groups for Improving Empowerment, Quality of Life, and Functioning of People With Severe Mental Illness: A Randomized Clinical Trial
Source: JAMA Psychiatry. 2021 Oct 13;78(12):1–11. doi: 10.1001/jamapsychiatry.2021.2880 (PMC8515257; doi:10.1001/jamapsychiatry.2021.2880)
Supplement: Supplement 1. — Trial Protocol [file jamapsychiatry-e212880-s001.pdf]

Research protocol

Medical Ethics Committee at VU University Medical Centre, Amsterdam

Title: “Resource Groups in the Netherlands: Effects, Costs and Meaning”
Date: 18th of September 2017

Principal Investigator: Hans Kroon, Ph.D., Professor and Head of Department of Reintegration and Community Care, Trimbos Institute, Utrecht, the Netherlands; Professor, Department of Social and Behavioral Sciences, Tranzo Scientific Center for Care and Welfare, Tilburg University, Tilburg, the Netherlands

Email: HKroon@trimbos.nl

Funding

This trial was supported by a grant from the Dutch foundation “Stichting tot Steun VCVGZ” (grant nr: 431), and by additional funding from collaborating mental health centres.

Trial sites

The control and experimental interventions will be tested in eight mental health centers, that each participate with at least community-based mental health teams: Altrecht (region Utrecht, 2 teams), Antes (Rotterdam, 3 teams), Arkin (Amsterdam, 2 teams), Bavo (Rotterdam, 2 teams), GGZ Centraal (region Utrecht, 2 teams), GGZ Breburg (region Brabant, 2 teams), Lentis (region Groningen, 3 teams), GGZ ingeest (Amsterdam, 2 teams), GGZ NHN (region North-Holland).

Advisory board

Cornelis L. Mulder, Professor and Head of Research Department of Psychiatry, Erasmus University Medical Center, Rotterdam, the Netherlands; Psychiatrist Parnassia Psychiatric Institute, Rotterdam, the Netherlands

Stynke Castelein, Professor and Head of Research Lentis Research, Lentis Psychiatric Institute, Groningen, the Netherlands; Professor and Head of Research Rob Giel Research Center, University of Groningen, University Medical Center Groningen, Groningen, the Netherlands; Professor Faculty of Behavioural and Social Sciences, Department of Clinical Psychology and Experimental Psychopathology, University of Groningen, Groningen, the Netherlands.

Philippe Delespaul, Professor School of Mental Health and NeuroSciences, Maastricht University, Maastricht, the Netherlands; Clinical Psychologist Mondriaan Mental Health Trust, Maastricht/Heerlen, the Netherlands.

Rene Keet, Director Department of Community Mental Health, GGZ Noord-Holland-Noord, Heiloo, the Netherlands

Jaap van Weeghel, Professor, Phrenos Centre of Expertise, Utrecht, the Netherlands; Professor Department of Social and Behavioral Sciences, Tranzo Scientific Center for Care and Welfare, Tilburg University, Tilburg, the Netherlands

Researchers

Cathelijn Diana Tjaden, M.Sc., Ph.D. student affiliated with the study
Wouter den Hollander, Ph.D., co-promotor and affiliated with the statistics of the study

SMALL CHANGES MADE TO THE ORIGINAL PROTOCOL dd. 6th June 2021

- **COVID-19:** Due to COVID-19, the last 50 assessments of the 18-months follow-up measurements were made by phone/videocall.
- **Sample size:** We planned to include 180 participants. Our power calculations showed that 133 was needed in order to take possible drop-out into account. Our final sample included 158 participants, but our drop-out was less than anticipated. Thus although our sample size was smaller, this did not had an effect on the power of the analysis.
- **Participating centers and teams:** We had planned to recruit patients in nine mental health care centers throughout the Netherlands, with each center agreeing on participating with two teams at the time of writing the protocol. However, one team declined to participate, and there were 3 centers that included an extra team. Thus, recruitment took place at nine mental health centers, with a range of 1 to 3 teams per center.
- **Included patients per team:** We expected to reach a mean of N = 10 participants per team with a range of 6 to 14. There were however two teams that only recruited two patients, and there was one team that included 18 patients. Our range of patients per team was hereby larger than expected.

BACKGROUND AND RATIONALE

The number of adults (18 to 65 years) with severe mental illness (SMI) in Dutch mental health care is estimated up to 160.000 (Delespaul et al., 2013). People with SMI not only have to cope with their psychiatric symptoms but also with serious impairments in psychosocial and community functioning. Indeed, the nationwide survey "Panel Psychisch Gezien" in the Netherlands in 2016 (N=1519) revealed that many report problems of loneliness (more than 80%) and not being part of the society (40%). Moreover, only 20% has paid employment (including social employment) (van Hoof et al., 2016). The impoverished social environment is also reflected by the finding that 76% of long-term psychiatric patients name their professional as the most important person in their life (Borge, Martinsen, Ruud, Watne & Friis, 1999). However, people with SMI tend to have the same needs in life as other citizens but they seem to be in a disadvantageous position to realize these (Couwenbergh, et al., 2014). Good collaboration and communication between the patient, family, community resources and professional care would offer a promising starting point for an improved social and community integration for people with SMI.

Moreover, in the current health policy in the Netherlands there is a growing awareness for the importance of involving patients in their own care. That is, an increasing tendency is observed towards a health care system in which people with SMI are stimulated to be in control of their own lives, to use their own strengths and capacities in creating a meaningful daily living and to participate in society according to their own needs and wishes (Van Hoof, Van Erp, Boumans & Muusse, 2014). This evolving movement in Dutch mental health care is also referred to as recovery-supporting mental health care. Importantly, in this context recovery refers to 'learning to live better in the face of mental illness' (Davidson, Tondora & Ridgway, 2012) and it comprises multiple dimensions. Next to the often used clinical recovery (remission of clinical symptoms); personal recovery and societal recovery are thought to be determinants in the process towards a meaningful living in which people with psychiatric symptoms can retain grip on one's life and participate in society (Dröes & Plooy, 2010). Empowerment is developing into one of the key elements in this renewed view on providing good mental health care. It has been argued that recovery can only be successful if one feels capable

to recover or- in other words- feels empowered (De Haan & Oude Bos, 2011). The concept can be defined as processes in which the person rediscovers his identity and self-esteem and “takes his life in his own hands” (Boevink, Kroon, Delespaul & Van Os, 2016). Empowerment is recognized both as an outcome by itself, as well as an intermediate step to long-term health status and disparity outcomes for the individual (Wallerstein, 2006).

A promising way to increase empowerment and social integration is using Resource Groups (RG) (Nordén, Malm & Norlander, 2012a). RG may offer a new structure in Dutch mental health care to implement both improved social and community integration and the recent developments around recovery and empowerment in clinical practice. In the RG method, significant others from both the informal (friends, family, relatives) and the formal (social workers, nurses, case manager, psychiatrist) support system are systematically engaged in the treatment by means of the resource group meetings. The method is built around the basic theme that patients themselves are the directors of the group and choose their own RG members, set the goals for their treatment and have a decisive impact on how the RG meetings are designed (Nordén, Eriksson, Kjellgren & Norlander, 2012b). This change in structure and patterns of mental health care is a crucial factor in the empowerment of the patient. Empowered patients working in effective collaboration with their RG, in turn, are thought to improve their own health outcomes in terms of increased well-being and improved social and community functioning. Moreover, this process could become an important force in controlling health care costs (Nordén et al., 2012a). Additionally, systematically involving the informal support system of patients and paying attention to their role and burden related to the disease of the patient might reduce their stress and improve their psychical well-being. In other words, the patient’s personalized network is also empowered. As a consequence, there will be more stability within the direct environment of a patient and a lower degree of expressed emotions (EE). This way, supportive environments that supplement professional care in a collaborative manner can be realized.

The RG method has its origin in the “Optimal Treatment” model (OT model), integrating biomedical, psychological and social strategies in the management of severe mental illnesses (Falloon & Fadden, 1993). The treatment is focused on home-based assertive case management integrated with pharmacotherapy, with family or individual psychoeducation, and social and problem solving skills training offered when indicated (Petersen, et al., 2005; Falloon, Held, Roncone & Laidlaw, 1998). Within the OT model, the “family unit in the community” appeared to be an important element and evolved into a new concept in the beginning of the 2000’s in Sweden: the ’Resource Group’ (Jonsson & Malm, 2002). Here, the RG was embedded within the Resource Group Assertive Community Treatment (RACT) program (Nordén, et al., 2012b). The RACT program is a person-centered flexible assertive community treatment approach placing its emphasis on empowerment and rehabilitation of the client, delivered through a novel mechanism: a resource group clinical microsystem for each patient (Malm, Ivarsson & Allebeck 2014). A clinical microsystem is defined as a small group of people (including health professionals, patients and families) who work together in a defined setting on a regular basis to create care (Nelson, Batalden & Godfrey, 2007).

The first RCT study on the effectiveness of RACT showed significant improved social function and consumer satisfaction in favour of RACT, when compared with a well-established best practice community-based program that combined antipsychotic medication, family interventions and social skills training (Malm, et al., 2003). The main clinically important difference between the two treatment programs was that in RACT the treatment team not only consisted of professionals, but also of the patients and their significant others in which the concept of “shared decision making” was

central in order to formalize the participation of the patients and their significant others in contributing to the care.

A meta-analysis including 17 studies and a total of 2263 psychotic patients on the effectiveness of the RACT program for psychotic patients showed impressive effect sizes. That is, the study revealed that participation in the RACT program resulted in improved (social skills) functioning, increased well-being as well as a reduction of symptoms when compared to CAU, with effect sizes of respectively d = .82, d = 0.88 and d = .72 (Nordén et al., 2012a). The combined measure for the 17 studies and the three outcome parameters yielded a large effect size (d = 0.80). A review summarizing the findings of eight RCT's concluded that the RACT program consisting of involving patients and their significant others by clinical microsystem resource groups yielded positive effects on symptoms, functioning and well-being for patients with psychoses and that the method may be of use for patients within the entire psychiatric spectrum (Malm, Lundin, Rydell, Nordén & Norlander, 2015).

Although the RACT program comprises various new elements, the Resource Group (RG) has been classified as a major key component (Jonsson & Malm, 2002; Nordén et al., 2012b). The crucial focus in the RG model is on involving patient themselves and their significant others in the treatment and the treatment goals and on reinforcement of the client's own self-confidence and ability (empowerment). This way, all available resources in and around the patient are mobilized and integrated in order to shape most effective care. However, no study has been performed on the added value of the RG method. To isolate the RG as the potential declarative mechanism of the promising results of the RACT program, further research is needed. Moreover, knowledge is lacking about the effectiveness for patients within the entire psychiatric spectrum as well as potential obstacles and cost-effectiveness of implementing the RG-method in Dutch Mental Health care. Therefore, the present study will test the effectiveness of the RG method, the implementation, costs and meaning in a randomized controlled trial (RCT) in the Netherlands for patients within the entire psychiatric spectrum.

AIMS

The primary objective of the present study is to investigate the effectiveness of the Resource Groups on patient outcomes compared to care as usual alone for people with severe mental illnesses. It is hypothesized that participation in the RG is associated with increased empowerment, improved well-being and enhanced social and community functioning, when compared to care as usual.

The secondary objective is to investigate whether care as usual with RG is more cost-effective than care as usual alone. The RG method is hypothesized to be more cost-effective on the long term than care as usual.

The third objective is to explore the implementation process of the RG in Dutch mental health care and the deeper meaning for patients; and to assess potential obstacles, bottlenecks and success factors by using a qualitative case-approach.

STUDY DESIGN

Setting

The study will take place within the context of Flexible Assertive Community Treatment (FACT) (Van Veldhuizen, 2007). Investigations of the RG method in other contexts are also desirable in the future,

but this is beyond the aims of the present study. FACT is a community based outpatient treatment and rehabilitation program, intended primarily for individuals with severe mental illness. A multidisciplinary team of professionals (psychiatrist, psychologist, nurses, social worker, etc.) provides integrated care and support for the patient. The care is provided both at the mental health centers and in the own environment of the patient. Participating teams are generic FACT teams at the cooperating mental health centers for people with SMI and early psychosis (both are in this proposal referred to as FACT-teams).

Design and Allocation

The study is a multicenter randomized clinical trial (MRCT), including 180 patients of nine Dutch mental health care centers.

An independent statistician of the Trimbos Institute will generate a computerized allocation list, stratified on mental health care centers and teams. That is, for each team of every mental health care center a separate allocation sequence will be generated by means of allocation blocks assuming a maximum of fourteen participants per team. Two sizes of allocation blocks are used (i.e. 2 and 4), yielding three possible cells (i.e.: 2x4 and 2x2; 1x4 and 5x2; 0x4 and 7x2). These cells in turn, are randomized to keep the allocation unpredictable for the different stakeholders (care-givers and researchers). In order to minimize the risk of imbalance between conditions, the ratio of these possibilities will be stratified on resp. 1:2:2. The allocation sequence will be stored with the independent statistician and is concealed from all researchers, caregivers, and participants. Once allocated, condition is passed on to the central researcher and local staff by email so that the local intake procedure can be continued. Further matching between patient and case manager is allowed and performed by the local staff of the FACT-team. At start of the baseline measurement (0 months), written informed consent is obtained. Further assessments take place 9 (T1) and 18 (T2) months after obtaining informed consent.. Local independent research assistants will carry out all assessments. Assessments comprise self-report questionnaires (administered on PC) and interviews, see Table 1 and Table 2. Given the nature of our study, blinding of participants and treating clinicians is not possible. The local research assistants however are blinded. To optimize similarity in assessments, research assistants use an extensive standardized study protocol and discuss process in regular telephonic and face-to-face meetings with the central researcher.

Moreover, a qualitative case analysis will be performed in order to assess the implementation process, identify specific helpful and non-helpful aspects of the used protocol and to gain insight into the meaning of the RG method for patients, group members and professional caregivers. To this end, 8 to 10 patients and their RG (including case manager) are asked to participate in a qualitative study and will be monitored for a substantial period of time using an embedded multiple case study design (Yin, 2014). The selection of patients is performed by asking whether the patient approves to be approached for the qualitative research at the end of the baseline measurement of the quantitative study. Then, a selection will be made by using the data from the baseline measurement and optional consult of the case-manager. Some variation in the selection will be pursued (in location, new patients or longer in care, size and composition of the resource group, moderate/good therapeutic working relation). The case selection takes place in two steps with an interval of several months. Based on the first interview experiences, new cases will be sought to ultimately get a good picture of the core and variety of the experiences in the resource groups (purposive sampling). Some main topics that will be investigated are: helpful and harmful aspects of the RG, relationships within the RG, privacy aspects within the group and supervision and

training of case managers. The central researcher and an experienced researcher of Trimbos-specialized in qualitative study designs- will perform this part of the study. Participation of this part of the study is voluntarily and independent of participation of the quantitative part. All participants (both patients and their RG-members) will receive a transcript of the interviews and the observations and are asked to verify whether their opinion is expressed correctly (member check).

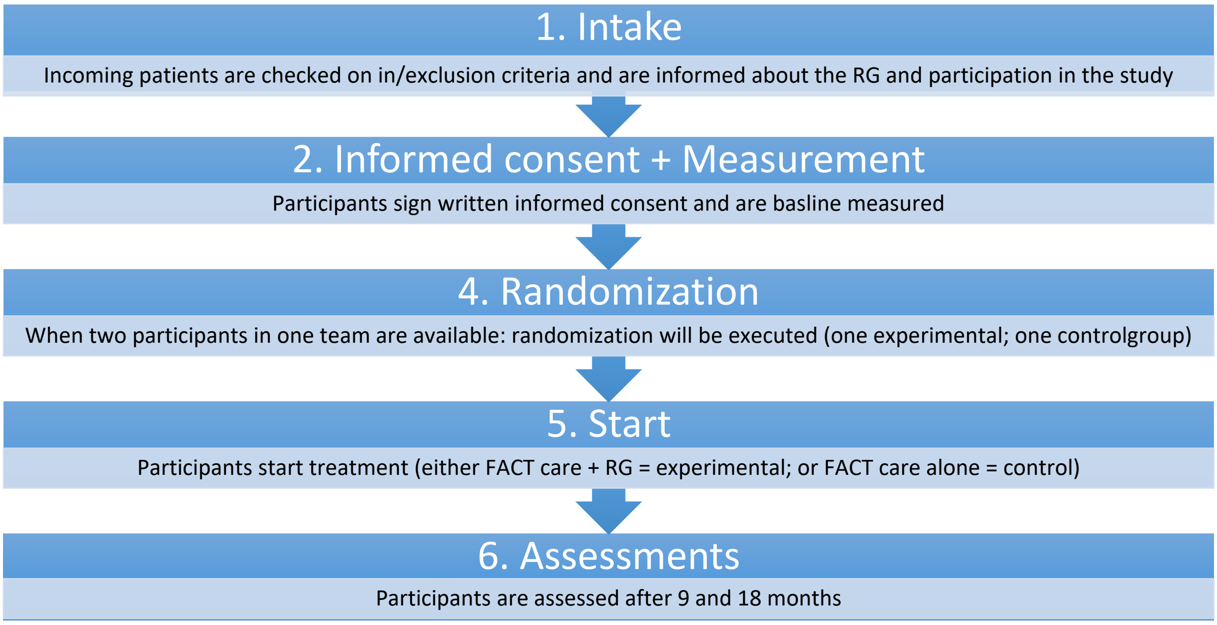

Fig 1. Randomization and assessment procedure

NB: if the incoming patients are not enough, patients who entered FACT care in the past 12 months will be screened on inclusion criteria and all will be informed about the RG. They will then follow the same procedure (starting with step 2).

STUDY POPULATION

Population

Patients meeting the criteria for SMI (Delespaul et al., 2013) that are indicated for generic FACT care will be recruited at the participating mental health centres. Male and female patients between 18 and 65 years old meeting the criteria for having a SMI will be included if they are expected to be in FACT care for the next 18 months and are able to understand and sign informed consent.

Inclusion criteria:

- Age between 18 – 65 years
- Having a severe mental illness: (1) a diagnosable mental, behavioral, or emotional disorder according to the diagnostic criteria specified within the DSM-5, (2) that is associated with serious social and/or societal limitations, (3) in which the limitation is the cause and effect of the psychiatric disorder, (4) in which the psychiatric disorder has a chronic course; and (5) for which coordinated care of mental health care professionals in care networks is indicated to realize the treatment plan (Delespaul et al., 2013).
- FACT-care for at least 18 months is indicated

Exclusion criteria

- Unable to give informed consent
- Insufficient knowledge of Dutch language

Sample size

Based on the meta-analysis of Nordén and colleagues (2012a), a pre-post effect size of Cohen's *d* = .5 for the primary outcome is expected. With a sample size of N=63 patients per condition, the study is powered at 80% to detect an effect size of Cohen's *d* = .5 at a two-tailed significance level of .05. With two conditions we would need to recruit N=126. Taking into account both repeated measures within a person, assuming a within-correlation of 0.6, and clustering of data (teams; assuming an ICC of .05, health centres; assuming an ICC of 0.1), we will need N=133 patients. Assuming a drop-out of 35%, we will recruit a total of N=180. There will be 18 participating teams over nine Dutch mental health centres (two teams at each site) and based on rates of incoming patients we expect that a mean of N=10 patients over the course of six months is feasible.

Recruitment

Patients are recruited in nine mental health care centers throughout the Netherlands that each agreed on participation with two teams (BAVO Europoort, GGZ NHN, GGZ inGeest, Lentis, GGZ Centraal, Antes, Breburg, Altrecht and Arkin). All incoming patients at the participating sites are checked for inclusion criteria by the local staff. Patients meeting the criteria are informed about the study and the RG during intake and asked to participate by the local intaker(s). If patients indicate to be interested, they will receive a patient information brochure and a week time to neutrally consider their participation. They will also be referred to the central researcher for any remaining questions, to ensure that they are fully informed about the nature of the study. Hereafter, the local staff of the FACT-team will contact the patient to inquire whether he/she wants to participate in the study. When the patient verbally agrees with participation the randomization is performed. To this end, the local staff sends a notification email to the central researcher with minimal contact information (surname and telephone number). The central researcher will perform the randomization by entering the following information to the generated allocation list: (1) name of the mental health care center; (2) name of the team; and (3) the sequential number of entry of the new participant. The local researcher will contact the patient to make the first appointment for the baseline measurement. Before start of the baseline measurement, participants sign written informed consent of which they will receive a copy.

In case the number of incoming patients is not enough, patients within the caseload of the trained casemanagers who entered FACT care in the past 12 months will be screened on inclusion criteria and all will be informed about the RG. They will follow the same procedure as described above. The aim is to include as much as possible participants per site, expecting to reach a mean of N=10 per site (ranging from 6 to 14). Recruitment will take place between September 2017 and September 2018, with follow up ending on September 2020.

For both inclusion and follow-up assessments, participants are contacted via the personal information they have provided and approved to use for the study. These entail surname and telephone number. They will be contacted through phone after which they can decide on the location to meet the researcher for the assessments.

INTERVENTION

Experimental group: FACT care as usual + Resource Groups

Patients will receive FACT care as usual. Next to this, they are guided to form their own Resource Group (RG) with whom they will meet on average once every three months. They are asked to prepare, attend and evaluate the RG meetings. The central element in the RG is the patient as the director of the group (Nordén et al., 2012b). That is, the patient nominates group-members sets his/her self-recovery treatment goals and decides on the location and time of the meetings, guided and supported by the case manager (CM). The CM and the patient always take part of every RG meeting, and the psychiatrist of the FACT-team attends the RG once a year if possible and necessary. Other RG-members can be significant others (family members, friends or others) as well as (family) experts and professionals from within and outside mental health care. The recovery goals can relate to all aspects of recovery, such as personal recovery (recovery of identity), social rehabilitation (meaningful participation and performance in society) and health (treating and improve physical and mental symptoms). During the meetings, the group decides on the role of each member in accomplishing the intended goal. The RG makes decisions together and takes responsibility for seeing to it that the development plan is followed (Nordén et al., 2012b). All the RG-meetings are clearly and consistently structured over time and follow the agenda that the patient and the CM agreed on. Additionally, an action plan for recognizing early warning signs that indicate an approaching relapse is established so that RG members can provide adequate, adjusted guidance that is designed and approved by the patient. Between the meetings and during evaluation, subjects are motivated to work on these goals and to involve the RG members in this process.

The CM coordinates the implementation of the RG and bears responsibility for monitoring that the RG method is carried out as intended. Moreover, the CM invites the nominated RG members for an interview before the first RG-meeting to explore the possible RG-participation as well as the relation between the nominated member and the patient. The purpose of these interviews is to give space to the story and the experiences of all RG-members. Also, there is attention to express possibly existing emotions towards the patient and/or other members of the RG so that the so called 'expressed emotions (EE)' are low during the RG meetings. Hereby, the CM has a key-role in creating a good emotional environment around the patient. Additionally, the CM can train the patient and the RG-members in- or outside the RG meetings when he/she notices structural problems within the group- for example emotional communication, handling stress, conflicts between members, inadequate knowledge about the mental disease or a lack of problem-solving skills, or suggest to invite a colleague into the RG to help with this. This way, maladaptive patterns can be addressed in order to remove the barriers that are blocking the way to a successful resource group. Alternatively, the CM can decide to involve an expert, for example in family therapy, for an extra session for (part of) the RG when more complex problems are evident.

Taken together, the CM facilitates a gradual transfer of personal responsibility to the client. That is, it is the client who is to be given influence and it is the task of the CM to give the client support and the tools to be able to take the power to carry that responsibility.

Comparison group: FACT care as usual

In the comparison group, patients will receive FACT care as usual. Importantly, patients in this group are also motivated to work on their treatment goals. Moreover, as is regular practice in FACT care, significant others are involved in the treatment and structural problems between patients and their significant others are addressed. However, these aspects are not to be handled according to the structure of the RG-method.

Case managers, training and model fidelity

At least two members from each FACT team receive an extensive 2-day training program before study start and two follow-up afternoons during the course of the study. Moreover, yearly booster sessions are organized. Two experienced and skilled trainers lead the interactive trainings. The program consists of lectures, role-plays and discussions to study and familiarize the vision, methodology and the content of the roles within the RG-approach. The central element during these days is to habituate on transferring the "guidance" to the patient and his chosen RG in order to nourish the patient's confidence to reach his or her own goals.

Adherence to the RG protocol is assessed using a RG model fidelity scale. Based on the RG handbook and Swedish material, the scale will be developed to estimate the extent to which the resource groups operate according the intended approach. A first draft of the scale will be presented to involved researchers, (peer)experts and representatives of the participating sites. After modifications, the scale is tested at two of the participating sites. Based on the experiences, the scale will be adjusted if necessary and the final version will be implemented at all sites. After 9 and 18 months, an external auditor will score all the RG's on the model fidelity together with the CM so that for each RG a model-fidelity score is obtained. Moreover, the control condition will be scored on model fidelity at 9 and 18 months. Part of the list is a logbook that case managers fill in after each RG-meeting (only for experimental condition). The logbook consists of 7-10 questions, answered online. The central researcher will go through the answers and can hereby assess (1) whether RG-meetings take place; and (2) whether the RG-meetings are accomplished according to the RG-method.

Moreover, trained case managers will attend peer-to-peer coaching sessions. These are telephone group sessions of 1h – 1.5h once every 8 weeks in a set group of max 8 case managers from different institutions/regions. The sessions will also be attended by the central researcher, allowing to keep track of recurrent themes and the quality of the session.

The central researcher will perform frequent visits to each participating team during the course of the study. The purpose of these work visits is to evaluate the RG approach in Dutch clinical practice and identify obstacles and improvements during implementation. Additionally, the trained case managers within a team meet once every two weeks with each other to discuss the different RG-group cases. Any recurrent questions/themes will be discussed with the central researcher, and if necessary with the trainers.

STUDY PARAMETERS (PRIMARY AND SECONDARY STUDY OUTCOMES)

Several questionnaires will be administered in order to assess recovery. To operationalize the concept of recovery, a three dimensional framework will be adapted (Couwenbergh et al., 2014):

1) *Personal recovery.* Defined as a highly personal process in which a patient can give meaning to previous events and steps are taken to regain grip at one's life (Stel, Stringer, & Koene, 2015). Personal recovery can be reached even if there are still (severe) clinical symptoms present (Wunderink, Nieboer, Wiersma, Sytema, & Nienhuis, 2013).
2) *Societal recovery.* Patients often experience social exclusion and broken relations with family and friends (Whitley & Drake, 2010). Societal recovery aims to degrade the public stigma on mental illness and improve the position and rights of (ex-)patients within society (Couwenbergh et al., 2014). Furthermore, it entails improvement in housing, work, education and social relations (Stel, 2012).

3) *Clinical recovery* is the traditional aim in mental health care, striving to decrease the presence of symptoms and to increase one's physical functioning (Huber et al., 2016).

Primary outcome parameter:

Personal recovery:

Empowerment will be assessed with the *'Nederlandse Empowerment Lijst'* (NEL; Boevink, Kroon, Delespaul & Os, van, 2017) containing 6 subscales: confidence and purpose, social support, connectedness, self-management, caring community and professional help. This questionnaire is selected because the domains closely match the outcomes that are expected from the RG-method. The list was established in collaboration with patients and experts-by-experience and is frequently used in evaluations inside and outside the Dutch Mental Health Care. Internal consistency (Cronbach's alpha = 0.94 total score; subscales ranging from 0.74 to 0.90), aspects of validity, reproducibility (intraclass correlation = 0.79) and responsiveness were good (Boevink et al., 2017). This was confirmed by Van Gestel-Timmermans and colleagues (2012). In trials, sensitivity to change has been shown (Van Gestel-Timmermans, Brouwers, Van Assen & Van Nieuwenhuizen, 2012; Michon, Wezep, van, Rijkaart, Overweg, Vink & Kroon, 2013).

Secondary outcome parameter:

Personal recovery

Quality of life will be assessed with the *Manchester Short Assessment of Quality of Life (MANSA; 16 items; Priebe et al., 1999).* The MANSA is a shortened version of the Lancashire Quality of Life Profile (LQLP; Oliver, 1991), and was specially developed for measuring quality of life in patients with psychological problems. The instrument consists of 16 items, including the satisfaction with work / unemployment, the quality of friendships and mental health on a seven-point scale (from "can not be worse" to "can not be better"). In addition, there are four questions answered with 'yes' or 'no', such as the presence of a good friend. The MANSA is completed by the client.

I.ROC. The Individual Recovery Outcomes Counter (I.ROC) is a twelve-item facilitated questionnaire and is found to be a valid and reliable measure of recovery in mental health (Monger et al., 2013). That is, the I.ROC showed high internal consistency. Exploratory factor analysis indicated a two-factor structure comprising intrapersonal recovery (factor 1) and interpersonal recovery (factor 2), explaining between them over 50% of the variance in I.ROC scores. The I.ROC significantly correlated with widely used existing instruments assessing both personal recovery and clinical outcomes (resp. Recovery Scale (RAS) and the Behaviour and Symptom Identification Scale (BASIS-32)).

Societal recovery

Basic demographics, family history, socio-economic status, lifetime psychiatric illness diagnosis and psychiatric history (including number of compulsory admissions) will be enquired. Moreover, several questions regarding education, work, social network and frequency and importance of social contact are included to obtain objective information regarding social and societal functioning. Also, the following questionnaires will be collected:

General, social and societal functioning will be assessed with the *WHODAS 2.0 - 36*, interview version (Üstün, Kostanjsek, Chatterji & Rehm, 2010). The WHODAS 2.0 - 36 consists of 36 items produces standard disability measures across six domains: Cognition, mobility, self-care, getting along, life

activities and participation (World Health, 2014). The WHODAS 2.0 - 36 items has shown good psychometric properties in clinic and rehabilitation samples (Garin et al., 2010). Moreover, the questionnaire is responsive to pick up changes in the functioning profiles over time (Üstuün et al., 2010).

Additionally, to measure general functioning, the *Global Assessment of Functioning (GAF)* will be scored by the interviewer in line with previous work on the RG (Malm et al., 2014). The GAF outcome measures are the DSM-IV split-GAF disability and split-GAF symptoms rating scales (scores range from 100 (extremely high functioning) to 1 (severely impaired)). Research has suggested the GAF as a valuable outcome indicator (Gaite et al., 2005; Pedersen & Karterud, 2012).

The individual's level of social and occupational functioning will be assessed with the *Social and Occupational Functioning Assessment Scale* (SOFAS; Goldman, Skodol & Lave, 1992), rated by the researcher. The SOFAS scale is similar to the frequently used GAF-score, but it only focuses at social and occupational functioning rather than also considering symptom severity.

Clinical Recovery

General psychopathological symptoms as an index of severity of syndromal disorders will be assessed with the *Brief Symptom Inventory – 18 items* (BSI-18; Derogatis 2001). The BSI- consisting of 53 items- was reduced to the BSI-18 to decrease the average completion time and to improve its structural validity (Derogatis, 2001). A total score over all items can be calculated representing general distress, which is highly correlated with the total score from the BSI (r = .90; Andreu et al., 2008; Durá et al., 2006). Moreover, the total score of the BSI-18 is sensitive to change, and captures smaller changes in symptomatology. Therefore, the BSI is considered as a suitable outcome to measure therapy effect (De Beurs and Zitman, 2006). Next to the total score, a dimension score on somatic complaints, depression and anxiety can be obtained with the BSI-18.

Mediating variable

Attachment Style will be assessed with the *Revised Adult Attachment Scale* (RAAS; Collins, 1996) in order to identify underlying models of attachment as a potential mediating variable. The RAAS has 18 items, divided into 3 sub-scales: trust, support and concern. The psychometric properties of this list range from moderate to good and are described in various articles (Collins, 1996; Tait, Birchwood, & Trower, 2004).

Additional

The experienced care and burden of the involved RG-members will be assessed with the *'Betrokken Evaluatie Schaal'* (BES; Schene & Van Wijngaarden, 1991). The questionnaire includes four domains (stress, worry, supervision, motivating) and a total score. Questions will be slightly modified in order to use the questionnaire for various mental health disorders- as has been done before (for example van Meijel & Dekker, 2002). The validity and reliability of the BES are satisfactory (Van Wijngaarden, 2003).

For confounding and mediating effects, we will collect information on gender, age, marital state, occupational status, duration of illness, medication use and treatment history.

The use and appreciation of family participation and the RG-method will be assessed by a self-developed treatment satisfaction list that will be filled in by participants, RG-members and professionals. The questionnaire will be divided in two parts. The first part is about generic aspects of family participation and will be assessed in both groups; the second part is about specific RG elements and will only be assessed in the experimental RG group. Central in this questionnaire will be the extent to which the received care in both groups meets the needs of participants and their environment. Themes that will be addressed are: satisfaction with help and support for psychological problems, personal recovery, social relations, daily activities, financial affairs; cooperation and coordination in help and support; support of informal support system; personalized help and support (important matters are addressed); experienced influence and to be taken seriously.

Economic Evaluation

Use of health services will be assessed with the *Trimbos and Institute of Medical Technology Assessment Cost Questionnaire for Psychiatry* (TIC–P; Hakkaart-van Roijen et al, 2002). With this questionnaire patients register the number of general practice visits, sessions with psychiatrists, hospital admissions, days-in-hospital, compulsory care, contact with teams/services, etc. In addition, the number of 'work loss' days (absenteeism from work) and the number of 'work cut-back' days (reduced efficiency at work while feeling ill) were also measured with help of the TIC–P. Also, information on relapses defined by intake on the DigiBoard of the FACT-team is collected.

Qaly's will be calculated with the *EuroQol EQ-5D-5L* scores, including the EQ-VAS (Herdman et al., 2011; Rabin & Charro, 2001). The EQ-5D-5L is used so that Dutch unit prices can be administered for the valuation of cost prices (as opposed to the often used SF-6D that uses British unit prices) (Oostenbrink, Koopmanschap & Rutten, 2002). Moreover, the ReQol questionnaire has the potential to calculate Qaly's but this process is still ongoing. When there is sufficient scientific support, the ReQoL will be used as an alternative to the EQ-5D-5 to calculate Qaly's.

**Parameters**		**Test**	**Time (min)**
**Primary outcome**	Personal	Netherlands Empowerment List (NEL) (in Dutch: Nederlandse Empowerment Lijst; self-rated)	15
**Secondary**	Personal	Recovering Quality of Life, short version 10 items (ReQoL) (self-rated)	5
	Societal	WHO-DAS 2.0 -12 (self-rated)	5-10
		Self-reliance matrix (ZRM) (in Dutch: Zelfredzaamheidsmatrix) (observer-rated)	10-15
		GAF/SOFAS (observer-rated)	5
		Demographic information on social network, social contact, societal functioning, part 2 (DEM_2) (self-rated)	10
	Clinical	Brief Symptom Inventory – 18 items (BSI-18) (self rated)	5-10
**Economic Evaluation**		Trimbos and Institute of Medical Technology Assessment Cost Questionnaire for Psychiatry (TIC-P) (self-rated)	10
		EuroQol EQ-5D-5L (self-rated)	3

**Mediating**		Revised Adult Attachment Scale (RAAS) (self-rated)	5-15
		Demographic questionnaire on age, marital status, housing, part 1 (DEM_1) (self-rated)	10
Significant others (filled in by informal support system)		*Betrokken Evaluatie Schaal (BES)*	*10*

Table 1. *Questionnaires/interviews that subjects will undergo in the course of the research.*

	0 (0months)	1 (9months)	2 (18months)
NEL	X	X	X
ReQoL	X	X	X
WHODAS	X	X	X
ZRM	X	X	X
GAF/SOFAS	X	X	X
DEM_1	X		
DEM_2	X	X	X
BSI-18	X	X	X
TIC-P	X	X	X
EQ-5D-5L	X	X	X
RAAS	X		
BES (*filled in by informal support system)*	X	X	X

Table. 2. *Overview of the procedures per assessment moment.*

STATISTICAL ANALYSES:

Effectiveness RG

Data will be analyzed according to the "intention to treat" principle, including all patients regardless of whether they drop out from treatment or not (StataSE 12.1). We will use Student's t-tests for continuous variables and Pearson chi-squared tests for categorical variables to analyze potential between-condition differences in baseline characteristics. Variables that show different distributions in the conditions, and are correlated with the results, are included in the analysis as a covariate. We will use pre-specified cut-off points of the RG model fidelity checklist to define good adherence.

Primary outcome (empowerment) and secondary outcome parameters are analyzed by means of multilevel mixed regression models with 4 levels: observations within persons, persons within teams and teams within centers. Baseline values of the parameters will be included in the model in order to control for regression to the mean. For categorical outcome variables, counts and in case of non-normal residuals, appropriate forms of mixed regression will be chosen (binomial,

Poisson, gamma, etc). Results are described in accordance with the CONSORT guidelines for randomized controlled trials (Schulz, Altman & Moher, 2010).

Economic Evaluation

The economic analysis is also performed according to the intention-to-treat principle. Both a cost-effectiveness analysis (CEA) (outcome NEL) and a cost-utility analysis (CUA) (effects expressed as QALY's, based on EuroQol utility score) will be done. Primary outcome parameters are the proportion of increased quality of life measures (CEA), and total QALY gained during 18 months (CUA). We will adapt a societal perspective, including the costs of all types of health services and the costs that stem from production losses. The time frame of this study is restricted to 18 months. Therefore, we will not correct for inflation and will not discount costs. All costs are expressed in euros (e) for the reference year 2016.

Robustness and uncertainty around the cost-effectiveness ratio (ICER) are determined by non-parametric bootstrapping. The point estimation of the ICER and the results of the bootstrap analyzes are graphically displayed in a cost-effectiveness plane in order to assess whether the RG appears to be acceptable from a cost-effectiveness perspective. Also, sensitivity analyses will be performed to test the robustness of the results. Some sensitivity analyses are: analyzing only data from study completers (patients who have complete data sets at all three assessments), analyzing costs when the more narrow perspective of direct costs is used (instead of the broader societal perspective), and correcting for baseline costs and utilities. Results are described in accordance with the CHEERS guidelines for economic health evaluations (Husereau et al., 2013).

Case-study

Content analyses are executed using the insights on case studies of Yin (2014) and the grounded theory approach (Glaser & Strauss, 1967). Elements of the analysis include making a case description at the resource group level, labeling, clustering and interpreting recurring patterns and themes, studying striving results and iteratively building a (declaration) model (Yin, 2014). In line with the grounded theory approach (Glaser & Strauss, 1967), the theory is derived from empirical data. To increase the validity, two persons jointly perform the case studies, and member checks are applied (hereby involving resource groups).

SUBJECT BURDEN

Subjects in both conditions are asked to participate in the three assessment appointments, 0, 9 and 18 months after giving informed consent. The assessments take 1,5 to 2 hours and consist of self-report questionnaires and interviews, guided by the research-assistant. When the research-assistant notices that this is too long for the participant (decreased concentration, fatigue, symptoms) the appointment may continue at another time. Data collection for RG-members (experimental condition) and significant others (control condition) is provided via mail with an online questionnaire. Moreover, in the experimental RG-condition subjects are asked to prepare, attend and evaluate the RG-meetings.

RISK ASSESSMENT SUBJECTS

A potential risk is the confrontation with possible (old) conflicts that may become apparent during the RG sessions. In order to minimize the risk, the case-manager will have interviews with all RG-members before the first meeting to make room for the story, difficulties and associated emotions of

the RG-members in relation to the patient. The intention of these interviews is to diminish the need to raise these issues during the meeting. Indeed, in the trainings of the case-managers and in the handbook it is explicitly instructed to aim for a constructive and safe working atmosphere for all RG members. Any themes or conflicts with high emotional intensity that threaten this atmosphere are to be handled outside the meetings.

Several mental health settings (e.g. BAVO, InGeest, GGZ NHN, Lentis) have performed pilots with the RG-method. These experiences indicate that the instructions are indeed adequate and do not reveal concerns about this particular risk nor have they identified other risks.

POTENTIAL ADVANTAGES PARTICIPATION

The potential value of the study is that we gain insight in the effectiveness of restructuring the provided mental health care for people with SMI. That is, by structurally and consistently involving significant others from both the informal and formal support system and centering the patient in his/her own care we expect that the provided care can be better tailored to the daily situation of the patient and his/her own needs and wishes.

Possible direct benefits of participating in this study is that we offer patients with SMI a program to feel in control of one's own treatment, to better integrate into society and to share the disease burden with significant others.

POTENTIAL DISADVANTAGES PARTICIPATION

A direct burden for the subjects is that they have to fill out several questionnaires. However, the interviewers are trained to establish a confidential, interpersonal relationship before starting the assessment in order to create an atmosphere in which participants feel comfortable enough to set their boundaries. Moreover, the population of interest is a group in which the themes of the selected questionnaires are discussed regularly during treatment so we don't expect that the content of the questions are new or significantly burdensome for the participants.

Secondly, the content of the treatment is not extensively affected by randomization. That is, in both conditions significant others can be involved into treatment and in both conditions patients and caregivers will work onto succeeding the treatment goals that are important for the patient. The main distinguishing difference between the conditions is the systematic way and the structure in which this is done. Moreover, all patients can withdraw from the RG and the study at any time, without consequences for the FACT treatment.

REIMBURSEMENT

Subjects will receive vouchers with a value of €15 (in Dutch: VVV-bon) for each of the assessment moments.

ADMINISTRATIVE ASPECTS

All the data will be handled and stored confidentially in compliance with the Dutch Personal Data Protection Act. Completed paper documents, such as consent forms and contact forms, are stored and secured. Contact- and personal information will be stored in a digital database separately from the research data. Participant codes will be used to link the research data with the identifiable information of the participant. These codes are not based on the participants' initials, birth-data or other participant related characteristics. The principal investigators are the only persons with access to the code file.

Electronic and printed research data necessary to reproduce analysis and quality checks will be kept for 10 years at Trimbos Institute conform local and official regulations. Identifiable information will be destroyed maximum 5 years after the end of the study.

PUBLICATION

The study is part of a dissertation, supported by a large project team consisting of leading professors, psychiatrist, researchers and experts-by-experience. The members will present the results of the study in their writings, presentations at conferences and during teaching. Moreover, the PhD student will present the goals and the results of this study at (inter) national conferences and meetings. The design of the study as well as the results will be submitted to international and Dutch peer-reviewed journals as part of the dissertation. None of the results (used or unused) will contain any personal information.

REFERENCES

Andreu, Y., Galdón, M. J., Durá, E., Ferrando, M., Murgui, S., García, A. & Ibáñez, E. (2008). *Psychometric properties of the Brief Symptoms Inventory–18 (BSI-18) in a Spanish sample of outpatients with psychiatric disorders.* Psicothema, 20, 844–850.

Boevink, W., Kroon, H., Delespaul, Ph., & Os. J. van (2017). Empowerment according to Persons with Severe Mental Illness: Development of the Netherlands Empowerment List and its Psychometric Properties. *Open Journal of Psychiatry, 7*(1). doi:10.4236/ojpsych.2017.71002

Borge, L., Martinsen, E. W., Ruud, T., Watne, O., & Friis, S. (1999). Quality of life, loneliness, and social contact among long-term psychiatric patients. *Psychiatric Services, 50,* 81-84.

Collins, N. L., & Read, S. J. (1990). Revised adult attachment scale. *Unpublished instrument, Department of Psychology, University of Southern California, Los Angeles, CA*.

Couwenbergh, C., Weeghel, J. van, Delespaul, P.A.E.G., Gaag, M. van der, Giesen, I. van der & Gool, R. van. (2014). *Over de brug.* Utrecht, Nederland: Phrenos.

Davidson, L., Tondora, J. & Ridgway, P. (2012) Life is not an 'outcome': reflections on recovery as an outcome and as a process. *Journal of Psychiatric Rehabilitation*. Vol. 13. No. 1. pp 1-8.

De Beurs, E. & Zitman F. De Brief Symptom Inventory (BSI) (2006). De betrouwbaarheid en validiteit van een handzaam alternatief voor de SCL-90. *Maandblad Geestelijke volksgezondheid, 61*: 120-41.

De Haan, G. & Oude Bos, J. (2011). *Veerkrachtig, weerbaar en zelfsturend. Op weg naar herstelondersteunende verslavingszorg.* Dalfsen/Groningen: Kennisnetwerk Het Zwarte Gat.

Delespaul, P. en de consensusgroep EPA (2013). Consensus over de definitie van mensen met een ernstige psychische aandoening (epa) en hun aantal in Nederland. *Tijdschrift voor psychiatrie 55*, 6.

Derogatis, L. R. (2001). *Brief Symptom Inventory (BSI)-18: Administration, scoring and procedures manual*. Minneapolis, MN: NCS Pearson.

Durá, E., Andreu, Y., Galdón, M. J., Ferrando, M., Murgui, S., Poveda, R. & Jimenez, Y. (2006). Psychological assessment of patients with temporomandipular disorders: Confirmatory analysis of the dimensional structure of the Brief Symptoms Inventory 18. *Journal of Psychosomatic Research, 60,* 365–370.

Dröes, J. & Plooy, A. (2010). Herstelondersteunende zorg in Nederland: vergelijking met Engelstalige literatuur. *Tijdschrift voor Rehabilitatie, 19*(2): 6-17.

Falloon, I.R.H., Fadden, G. (1993). *Integrated Mental Health Care. A community based approach.* Cambridge, Engeland: Cambridge University.

Falloon, I.R.H., Held, T., Roncone, R. & Laidlaw, T.M. (1998). Optimal treatment strategies to enhance recovery from schizophrenia. *ANZ J Psychiatry, 32*: 43–49.

Fassaert, T., Lauriks, S., van de Weerd, S., Theunisen, J., Kikkert, M., Dekker, J., …. Wit, M. de. (2014). *Community Ment Health Journal, 50:* 583. doi:10.1007/s10597-013-9683-6.

Gaite, L., Vázquez-Barquero, J.L., Herrán, A., Thornicroft, G., Becker, T., Sierra-Biddle, D., Ruggeri, M., Schene, A., Knapp, M., Vázquez-Bourgon, J., EPSILON Group (2005). Main determinants of global assessment of functioning score in schizophrenia: a European multicenter study. *Comprehensive Psychiatry*; 46: 440–446.

Garin, O., Ayuso-Mateos, J. L., Almansa, J., Nieto, M., Chatterji, S., Vilagut, G., … Ferrer, M. (2010). Validation of the "World Health Organization Disability Assessment Schedule, WHODAS-2" in patients with chronic diseases. *Health and Quality of Life Outcomes*, *8*, 51.

Goldman, H.H., Skodol, A.E. & Lave, T.R. (1992). Revising Axis V for DSM-IV: A Review of Measures of Social Functioning. *American Journal of Psychiatry 149:1148–1156.*

Hakkaart-Van Roijen, L., Van Straten, A., Donker, M., *et al* (2002) *Manual: Trimbos/iMTA Questionnaire for Costs Associated with Psychiatric Illness* (in Dutch). Rotterdam: Erasmus University.

Herdman, M., Gudex, C., Lloyd, A., Janssen, M. F., Kind, P., Parkin, D., ... & Badia, X. (2011). Development and preliminary testing of the new five-level version of EQ-5D (EQ-5D-5L). *Quality of Life Research*, 20(10), 1727-1736.

Huber, M., van Vliet, M., Giezenberg, M., Winkens, B., Heerkens, Y., Dagnelie, P. C., & Knottnerus, J. A. (2016). Towards a 'patient-centred' operationalisation of the new dynamic concept of health: a mixed methods study. *BMJ Open, 6*(1).

Husereau, D., Drummond, M., Petrou, S., Carswell, C., Moher, D., Greenberg, D……..& Loder, E.; CHEERS Task Force (2013). Consolidated Health Economic Evaluation Reporting Standards (CHEERS) statement. *BMC Medicine* 11:80.

Jonsson, J., & Malm, U. (2002) The social network resource group in Sweden: a major ingredient for recovery in severe mental illness. In *Family Interventions in Mental Illness*, H.P. Lefley HP & D. Johnson (eds.). Westport Conn: Praeger Publications.

Keetharuth, A., Brazier, J., Connell, J., Carlton, J., Buck, E. T., Ricketts, T., & Barkham, M. (2017). *Development and Validation of the Recovering Quality of Life (ReQoL) Outcome Measures.* Derived from: http://www.eepru.org.uk/EEPRU%20report%20ReQoL%20validation%20v0.20.pdf

Malm, U.I., Ivarsson, B.A.R. & Allebeck, P. (2014). Durability of the Efficacy of Integrated Care in Schizophrenia: A Five-Year Randomized Controlled Study. *Psychiatric Services, 65(8).* 1054 1057.

Malm, U.I., Lundin, L., Rydell, P., Nordén, T. & Norlander, T. (2015). Resource Group ACT (RACT) – A Review of an Integrative Approach to Psychoeducation of Individual Families Involving the Patient. *International Journal of Mental Health 44(4). 269-276.*

Meijel, B. van & M.M. Dekker (2002). *Onderzoeksprogramma Verplegingswetenschap. Zelfmanagement van de chronisch zieke en zijn familie.* Utrecht: Academie Gezondheidszorg Utrecht.

Michon, H., Wezep, M. van, Rijkaart, A.M., Overweg, K., Vink, L. & Kroon, H. (2013). *Bewijs gevonden. Kwetsbare vrijwilligers sterker met Erkenning Verworven Competenties – Algemene vrijwilligerscompetenties.* Utrecht: Movisie.

Nelson E.C., Batalden P.B. & Godfrey M.M. (2007). *Quality by Design: A Microsystems Approach.* San Francisco: Jossey-Bass, 2007.

Nordén, T., Eriksson, A., Kjellgren, A. & Norlander, T. (2012b). Involving clients and their relatives and friends in the psychiatric care. Case managers' experiences of training in Resource group Assertive Community Treatment. *PsyCh Journal; 1:* 15-27.

Nordén, T., Malm, U., & Norlander, T. (2012a). Resource Group Assertive Community Treatment (RACT) as a Tool of Empowerment for Clients with Severe Mental Illness: A Meta-Analysis. *Clinical Practice and Epidemiology in Mental Health : CP & EMH, 8,* 144–151.

Oostenbrink, J. B., Koopmanschap, M. A. & Rutten, F. F. H. (2002) Standardisation of costs: the Dutch manual for costing in economic evaluations. *Pharmacoeconomics*, 20, 443– 454.

Pedersen, G. & Karterud, S. The symptom and function dimensions of the Global Assessment of Functioning (GAF) scale. *Comprehensive Psychiatry 53*: 292–298.

Petersen, L., Jeppesen, P., Thorup, A., Abel, M.B., Ohlenschlaeger, J., Christensen, T.O. et al. (2005). A randomised multicentre trial of integrated versus standard treatment of patients with a first episode of psychotic illness. *BMJ*: 331-602.

Rabin, R., & Charro, F. D. (2001). EQ-SD: a measure of health status from the EuroQol Group. *Annals of Medicine*, *33*, 337-343.

Schene, A. & B. van Wijngaarden (1993). Familieleden van psychotische patiënten, een onderzoek onder Ypsilon-leden. *Maandblad voor geestelijke volksgezondheid, 48 (9)*, pp. 899-914.

Schulz, K.F., Altman, D.G. & Moher, D. (2010). CONSORT 2010 Statement: updated guidelines for reporting parallel group randomised trials. *BMJ, 340.* 332.

StataCorp. 2011. *Stata Statistical Software: Release 12*. College Station, TX: StataCorp LP.

Stel, J. C. v. d. (2012). *Focus op persoonlijk herstel bij psychische problemen*: Boom Lemma uitgevers

Stel, J. C. v. d., Stringer, B., & Koene, J. (2015). *Perspectief op Potenties. Herstel door versterken executieve functies en zelfregulatie.* Retrieved from

Tait, L., Birchwood, M., & Trower, P. (2002). A new scale (SES) to measure engagement with community mental health services. *Journal of Mental Health, 11*(2), 191-198.

Üstün, T. B., Kostanjsek, N., Chatterji, S., & Rehm, J. (2010). *Measuring health and disability: manual for WHO Disability Assessment Schedule (WHODAS 2.0)*. Geneve, Switzerland: World Health Organization.

Van Gestel-Timmermans, H., Brouwers, E.P., van Assen, M.A. & van Nieuwenhuizen, C. (2012). Effects of a peer-run course on recovery from serious mental illness: a randomized controlled trial. *Psychiatric Services 63(1):* 54-60.

Van Hoof, F., van Erp, N., Boumans, J. & Muusse, C. (2014). *Persoonlijk en maatschappelijk herstel van mensen met ernstig psychiatrische aandoeningen. Ontwikkelingen in praktijk en beleid.* Utrecht: Canon Nederland BV

Van Hoof, F., Knispel, A., Hulschbosch, L., Place,C., Muusse, C., Van Vugt, M. …….& Kroon, H. (2016). *Landelijke Monitor Ambulantisering en Hervorming Langdurige GGZ.* Utrecht: Trimbos Instituut.

Van Veldhuizen, J.R. (2007). FACT: a Dutch version of ACT. *Commun Ment health J (43).* 421-433.

Van Wijngaarden, B. (2003). *Consequences for caregivers of patients with severe mental illness: The development of the Involvement Evaluation Questionnaire.* Amsterdam: Universiteit van Amsterdam.

Wallerstein, N. (2006*). What is the evidence on effectiveness of empowerment to improve health?* Copenhagen, WHO Regional Office for Europe (Health Evidence Network report; http://www.euro.who.int/Document/E88086.pdf, accessed 01 February 2006).

Whitley, R., & Drake, R. E. (2010). Recovery: A dimensional approach. *Psychiatric Services, 61*, 1248 - 1250.

Wunderink, L., Nieboer, R. M., Wiersma, D., Sytema, S., & Nienhuis, F. J. (2013). Recovery in remitted first-episode psychosis at 7 years of follow-up of an early dose reduction/discontinuation or maintenance treatment strategy: long-term follow-up of a 2-year randomized clinical trial. *JAMA Psychiatry, 70*(9), 913-920.

Yin, R. (2014). Case Study Research: Design and Methods. (5th Edition). Thousand Oaks, CA: Sage.
