# Supplementary material for: Effectiveness of Resource Groups for Improving Empowerment, Quality of Life, and Functioning of People With Severe Mental Illness: A Randomized Clinical Trial
Source: JAMA Psychiatry. 2021 Oct 13;78(12):1–11. doi: 10.1001/jamapsychiatry.2021.2880 (PMC8515257; doi:10.1001/jamapsychiatry.2021.2880)
Supplement: Supplement 2. — eTable 1. The 6 Phases of the Resource Group Method eTable 2. Description and Criteria of Scores on the Resource Group Model Evaluation Tool eTable 3. Frequency, Mean and SD of Scores on Items of the Resource Group Model Evaluation Tool eTable 4. Outcomes Linear Mixed Model Sensitivity Analyses of Primary and Secondary Outcomes of Participants With ≥2 RG Meetings eTable 5. Outcomes Mediation Analyses eTable 6. The Netherlands Empowerment List (NEL) eFigure 1. Trajectory of Mean Empowerment Scores Over Time, Based on the Intention-to-Treat Population eFigure 2. Absolute Empowerment Scores Within Participants Across Time Points [file jamapsychiatry-e212880-s002.pdf]

Supplementary Online Content

Tjaden C, Mulder CL, den Hollander W, et al. Effectiveness of resource groups for improving empowerment, quality of life, and functioning of people with severe mental illness: a randomized clinical trial. *JAMA Psychiatry*. Published online October 13, 2021. doi:10.1001/jamapsychiatry.2021.2880

eTable 1. The 6 Phases of the Resource Group Method
eTable 2. Description and Criteria of Scores on the Resource Group Model Evaluation Tool
eTable 3. Frequency, Mean and SD of Scores on Items of the Resource Group Model Evaluation Tool
eTable 4. Outcomes Linear Mixed Model Sensitivity Analyses of Primary and Secondary Outcomes of Participants With ≥2 RG Meetings
eTable 5. Outcomes Mediation Analyses
eTable 6. The Netherlands Empowerment List (NEL)
eFigure 1. Trajectory of Mean Empowerment Scores Over Time, Based on the Intention-to-Treat Population
eFigure 2. Absolute Empowerment Scores Within Participants Across Time Points

This supplementary material has been provided by the authors to give readers additional information about their work.

© 2021 Tjaden C et al. *JAMA Psychiatry.*

eTable 1. The 6 Phases of the Resource Group Method

Phase	Actions
Preparation	Patient and case-manager draft sociogram;<br>Patient and case-manager nominate members for the RG;<br>Patient and case-manager draft the RG plan
Investment	Case-manager establishes contact with nominated significant others;<br>Case-manager interviews nominated significant others, covering at minimum:<br>• Their expectations, commitment and responsibility in the RG<br>• Their relationship and previous experiences with the patient and other nominated members
Planning	Patient and case-manager set date of first meeting;<br>Patient and case-manager set up and print agenda;<br>Patient decides on:<br>• The location<br>• The chairman<br>• The frequency
First meeting	All RG members introduce themselves or are introduced by the patient;<br>The patient and/or case-manager give a short explanation on the RG method and confidentiality;<br>The RG discusses the agenda:<br>• The recovery goals (both long term and short term)<br>• The crisis plan<br>• The role of each member, concrete actions to achieve the goals
Follow-up meetings	During the follow-up meetings:<br>• The RG evaluates goals, assignments and progress<br>• The RG updates the goals and the recovery plan, and decides on new actions to achieve the goals<br>• Skills trainings are available for RG members (e.g., problem solving and emotional communication)<br>The composition of the RG can change if different persons are better suited to achieve the updated goals;<br>Once a year the psychiatrist attends the meeting
Reorientation	Discussion on composition of the RG, depending on the phase of care (e.g. transition to general practitioner/social domain or to only informal RG members possible)

Abbreviation. RG = resource group

© 2021 Tjaden C et al. *JAMA Psychiatry.*

eTable 2. Description and Criteria of Scores on the Resource Group Model Evaluation Tool

Item	Description	Score				
		1	2	3	4	5
1	The minimum number of RG meetings during the study is 4.	0<br>meetings	1<br>meeting	2<br>meetings	3<br>meetings	4<br>meetings
2	Integration is achieved, when:<br>1. The psychiatrist attends the RG at least yearly<br>2. The degree of perceived support from the FACT team is > 3 is on a scale of 5.<br>3. The RG-meeting is discussed with the FACT team.<br>4. The treatment plan is discussed and established annually in the RG.	None of the criteria have been met	1 criterion has been met	2 criteria have been met	3 criteria have been met	4 criteria have been met
3	Preparatory in-depth interviews have been held with informal RG members.	With 0% of the informal RG members	With 25% of the informal RG members	With 50% of the informal RG members	With 75% of the informal RG members	With 100% of the informal RG members
4	Agency is facilitated if patients have (co)decided on the following points:<br>1. Recovery goals<br>2. Agenda of the meeting<br>3. Composition of the RG<br>4. Chairman<br>5. Location<br>6. Frequency	2<br>points	3<br>points	4<br>points	5<br>points	6<br>points
5	A complete RG plan consist of the following points:<br>1. Short term goals<br>2. Long term goals<br>3. Agreements for the RG per goal<br>4. Crisisplan	0<br>points	1<br>point	2<br>points	3<br>points	4<br>points

© 2021 Tjaden C et al. *JAMA Psychiatry.*

6	A RG-meeting is complete, when the RG covered the following points:<br>1. Short term goals<br>2. Long term goals<br>3. Agreements for the RG per goal<br>4. Crisisplan<br>5. Skill training for the RG members	1<br>point	2<br>points	3<br>points	4<br>points	5<br>points
7.	The mean score of expressed emotions during the RG meeting is determined by scores on:<br>1. Hostility<br>2. Critics<br>3. Overinvolvement<br>4. Warmth (reversed)<br>5. Positive comments (reversed)	Mean score between:<br>8 - 10	Mean score between:<br>6- 8	Mean score between:<br>4 - 6	Mean score between:<br>2 - 4	Mean score between:<br>0 -2

Abbreviation. RG = resource group

© 2021 Tjaden C et al. *JAMA Psychiatry.*

eTable 3. Frequency, Mean and SD of Scores on Items of the Resource Group Model Evaluation Tool[a]

Item[b]	Frequency					Mean (SD)
	1	2	3	4	5	
1	0	15	11	7	26	3.75 (1.27)
2	0	4	26	22	7	3.54 (0.80)
3	4	1	14	5	35	4.03 (1.31)
4	0	3	11	13	32	4.25 (0.94)
5	0	2	1	7	49	4.75 (0.66)
6	0	7	21	24	7	3.52 (0.86)
7	0	0	6	19	34	4.07 (0.83)

[a]Data were collected after each RG meeting; RGs without a RG meeting are therefore not included. Additionally, the amount of RG meetings and thereby data of the scores per RG could differ.
[b]Description of items and scores can be found in eTable 2.

Abbreviation. RG = resource group

© 2021 Tjaden C et al. *JAMA Psychiatry.*

eTable 4. Outcomes Linear Mixed Model Sensitivity Analyses of Primary and Secondary Outcomes of Participants With ≥2 RG Meetings

Outcome and Effect	Slope (SD)	Cohen *d* effect size [95%CI][a]
**PRIMARY OUTCOME**		
Empowerment (NEL)[a]		0.61 [0.28 to 0.93]
Control Group	0.031 (0.004)	
Treatment Group	0.499 (0.005)	
**SECONDARY OUTCOMES**		
Quality of Life (MANSA)[b]		0.32 [0 to 0.64]
Control Group	0.187 (0.007)	
Treatment Group	0.541 (0.011)	
Personal recovery (I.ROC)[c]		0.47 [0.15 to 0.79]
Control Group	0.268 (0.007)	
Treatment Group	0.781 (0.011)	
Disability (WHODAS 32)[d]		-0.42 [-0.1 to -0.74]
Control Group	-2.599 (2.924)	
Treatment Group	-11.663 (4.365)	
General functioning (GAF)[e]		0.33 [0.01 to 0.65]
Control Group	3.435 (1.522)	
Treatment Group	8.541 (2.168)	
Social and occupational functioning (SOFAS)[f]		0.35 [0.03 to 0.66]
Control Group	2.012 (1.513)	
Treatment Group	7.328 (2.148)	
Symptoms (BSI)[g]		0.07 [-0.24 to 0.39]
Control Group	-0.216 (0.016)	
Treatment Group	-0.099 (0.023)	
Attachment unsafety (RAAS)[h]		0.14 [-0.17 to 0.45]
Control Group	-0.057 (0.004)	
Treatment Group	-0.178 (0.007)	
Frequency social contact[i]		0.07 [-0.24 to 0.39]
Control Group	-0.216 (0.016)	
Treatment Group	-0.099 (0.023)	
Quality social contact[j]		0.26 [-0.06 to 0.57]
Control Group	0.035 (0.005)	
Treatment Group	0.261 (0.007)	
Employment[k]		0.19 [-0.13 to 0.5]
Control Group	-0.028 (0.000)	
Treatment Group	0.877 (0.000)	

© 2021 Tjaden C et al. *JAMA Psychiatry.*

Abbreviations: NEL, Netherlands Empowerment List; MANSA, Manchester Short Assessment of Quality of Life; I.ROC, Individual Recovery Outcomes Counter; WHODAS, World Health Organization Disability Assessment Schedule 2.0; GAF, Global Assessment of Functioning Scale; SOFAS, Social and Occupational Functioning Scale; BSI, Brief Symptom Inventory; RAAS, Revised Adult Attachment Scale.
[a]Scores range from 1 to 5, with higher scores indicating better empowerment
[b]Scores range from 1 to 7, with higher scores indicating better quality of life
[c]Scores range from 1 to 6, with higher scores indicating better recovery
[d]Scores range from 1 to 5, with higher scores indicating more disability
[e]Scores range from 0 to 100, with higher scores indicating better functioning
[f]Scores range from 0 to 100, with higher scores indicating better social functioning
[g]Scores range from 1 to 5, with higher scores indicating more symptoms
[h]Scores range from 1 to 5, with higher scores indicating more attachment unsafety
[i]Scores range from 1 to 7, with higher scores indicating higher frequency
[j]Scores range from 1 to 7, with higher scores indicating better quality of social contact
[k]Zero indicates no job; 1, having a volunteer or paid job.

eTable 5. Outcomes Mediation Analyses

	Total Effect	AMCE [a]	ADE [b]	Proportion Mediated
Quality of Life (MANSA)				
Overall B (95%CI) [c]	0.30 (-0.04 to 0.67)	0.18 (0.01 to 0.38)*	0.12 (-0.19 to 0.44)	0.56 (-2.04 to 3.53)
N	119	119	119	119
Personal Recovery (I.ROC)				
Overall B (95%CI) [c]	0.57 (0.25 to 0.89) ***	0.19 (0.01 to 0.38)*	0.38 (0.10 to 0.67)**	0.32 (0.03 to 0.70)*
N	119	119	119	119
Disability (WHODAS - 32)				
Overall B (95%CI) [c]	-0.27 (-0.46 to -0.05)	-0.07 (-0.16 to 0.00)	-0.19 (-0.39 to 0.00)	0.26 (-0.02 to 0.88)
N	119	119	119	119
General Functioning (GAF)				
Overall B (95%CI) [c]	10.05 (5.31 to 15.07)	1.39 (0.06 to 3.40)	8.67 (3.84 to 13.48)	0.13 (0.01 to 0.36)
N	119	119	119	119
Social and Occupational Functioning (SOFAS)				
Overall B (95%CI) [c]	9.06 (3.63 to 13.22)	1.16 (-0.03 to 2.88)	6.90 (2.41 to 11.99)	0.13 (-0.01 to 0.41)
N	119	119	119	119
Symptoms (BSI)				
Overall B (95%CI) [c]	-0.08 (-0.36 to 0.18)	-0.14 (-0.29 to 0.00)	0.05 (-0.19 to 0.28)	0.80 (-6.87 to 15.02)
N	119	119	119	119
Attachment unsafety (RAAS)				
Overall B (95%CI) [c]	-0.09 (-0.31 to 0.13)	-0.07 (-0.16 to 0.00)	-0.02 (-0.23 to 0.18)	0.44 (-5.21 to 6.78)
N	119	119	119	119
Frequency social contact				

© 2021 Tjaden C et al. *JAMA Psychiatry.*

Overall B (95%CI) [c]	0.36 (-0.06 to 0.79)	0.10 (-0.00 to 0.25)	0.26 (-0.17 to 0.70)	0.24 (-1.23 to 1.80)
N	119	119	119	119
Quality social contact				
Overall B (95%CI) [c]	0.24 (-0.01 to 0.50)	0.10 (0.01 to 0.20)	0.15 (-0.08 to 0.39)	0.38 (-0.31 to 2.12)
N	119	119	119	119
Employment				
Overall B (95%CI) [c]	0.17 (-0.03 to 0.37)	0.01 (-0.03 to 0.06)	0.16 (-0.05 to 0.36)	0.04 (-0.56 to 0.84)
N	119	119	119	119

Note. [a] ACME = average causal mediation effects; [b] ADE = average direct effects; [c] B = standardized mean difference, CI = Confidence Interval

Abbreviations: NEL, Netherlands Empowerment List; MANSA, Manchester Short Assessment of Quality of Life; I.ROC, Individual Recovery Outcomes Counter; WHODAS, World Health Organization Disability Assessment Schedule 2.0; GAF, Global Assessment of Functioning Scale; SOFAS, Social and Occupational Functioning Scale; BSI, Brief Symptom Inventory; RAAS, Revised Adult Attachment Scale.

* p < .05
** p< .001

© 2021 Tjaden C et al. *JAMA Psychiatry.*

eTable 6. The Netherlands Empowerment List (NEL)					
**<u>At this moment</u>…….**	**1= Strongly disagree**	**2= Disagre**	**3= Neutral**	**4 = Agree**	**5 = Strongly agree**
1. My caregiver and I have a good collaborative relationship					
2. This society makes allowance for people with a psychiatric disability					
3. I have the feeling that I can mean something for someone else					
4. I have a purpose in my life					
5. The people around me accept me					
6. My caregiver takes my abilities as a starting point, not my limitations					
7. I am able to set my boundaries					
8. Those around me offer me a listening ear					
9. The people around me take me as I am					
10. I know what is good and what is not good for me					
11. In our society, people with a mental disability are considered full citizens					
12. I derive satisfaction from the things that go well					
13. I am able to deal with the problems that come my way					
14. My caregiver is there for me when I need him/her					
15. I decide how I control my life					
16. The people I love support me					
17. I can obtain adequate support when I need it					
18. I am determined to go on					
19. Society respects my rights as a citizen					
20. I have structure in my life					
21. The role of patient is no longer central in my life					
22. I am not afraid to ask for help					
23. This society offers social security to people with a mental disability					
24. I regularly meet people outside my home					
25. I can share my experiences with others with similar experiences					
26. I know what I am good at					
27. I have a good relationship with the people around me					
28. This society creates opportunities that fit my level of participation					
29. The care I receive fits in well with my life					
30. I have a sense of belonging					
31. I think of myself as a person worth something					
32. I turn negative thoughts into positive ones					
33. I can see how my life has made me who I am today					
34. I find peace and safety in my home					
35. I have enough to do each day					
36. This society does not discriminate against people with a mental disability					
37. I do the things that I think are important					
38. I can deal with my vulnerabilities					
39. I can fall back on the people around me					
40. I am not afraid to rely on myself					

© 2021 Tjaden C et al. *JAMA Psychiatry.*

eFigure 1. Trajectory of Mean Empowerment Scores Over Time, Based on the Intention-to-Treat Population

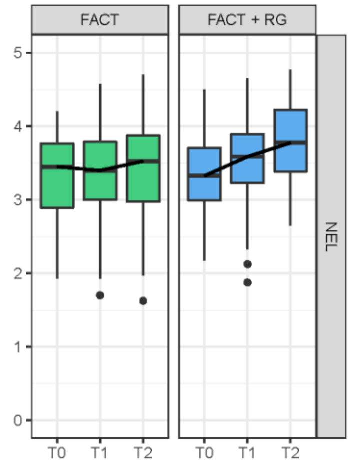

Error bars indicate the standard errors. FACT indicates care-as-usual; FACT + RG, resourcegroups integrated in care-as-usual. T0 represents baseline scores; T1 the 9-month follow-up and T2 the 18 month follow-up.

© 2021 Tjaden C et al. *JAMA Psychiatry.*

eFigure 2. Absolute Empowerment Scores Within Participants Across Time Points

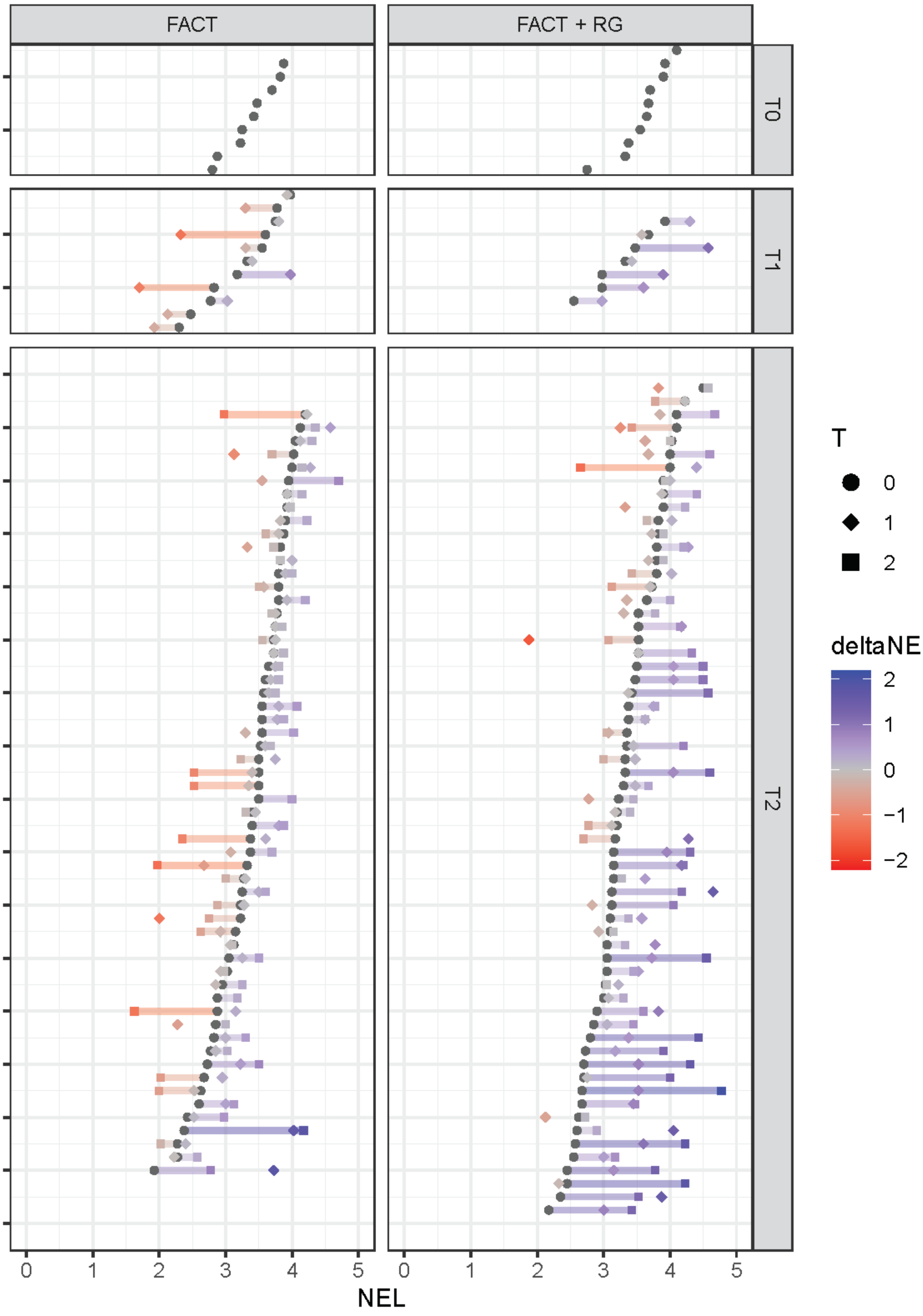

Participants are sorted by empowerment score [Netherlands Empowerment List; NEL:] at T0 [baseline] per pane. Left panes show participants in the FACT alone [care-as-usual] condition, and right panes show participants in the FACT + RG [RGs integrated in care-as usual]. The upper pane contains the participants that completed only T0; the middle pane contains the participants that completed T0 and T1 [9-months follow-up] and the lower pane contains the participants that completed T0, T1 and T2 [18-months follow-up]. In the middle and lower panes coloured lines are drawn within participants from T0 to the latest measurement. Colours indicate the direction and extent of change (deltaNEL).

© 2021 Tjaden C et al. *JAMA Psychiatry.*
