# Supplementary material for: Effectiveness of Resource Groups for Improving Empowerment, Quality of Life, and Functioning of People With Severe Mental Illness: A Randomized Clinical Trial
Source: JAMA Psychiatry. 2021 Oct 13;78(12):1–11. doi: 10.1001/jamapsychiatry.2021.2880 (PMC8515257; doi:10.1001/jamapsychiatry.2021.2880)
Supplement: Supplement 3. — Data Sharing Statement [file jamapsychiatry-e212880-s003.pdf]

Data Sharing Statement

Tjaden. Effectiveness of Resource Groups for Improving Empowerment, Quality of Life, and Functioning of People With Severe Mental Illness. *JAMA Psychiatry*. Published October 13, 2021. doi:10.1001/jamapsychiatry.2021.2880

Data
Data available: Yes
Data types: Deidentified participant data
How to access data: hkroon@trimbos.nl
When available: With publication

Supporting Documents
Document types: Statistical/analytic code
How to access documents: https://www.se80.co.uk/
When available: With publication

Additional Information
Who can access the data: researchers who provide a methodologically sound proposal to achieve aims in the approved proposal
Mechanisms of data availability: with a signed data access agreement with the Trimbos Insitute (Utrecht, the Netherlands)
Any additional restrictions: none
